# Exosomes from TNF-*α*-treated human gingiva-derived MSCs enhance M2 macrophage polarization and inhibit periodontal bone loss

**DOI:** 10.1016/j.actbio.2020.12.046

**Published:** 2020-12-24

**Authors:** Yuki Nakao, Takao Fukuda, Qunzhou Zhang, Terukazu Sanui, Takanori Shinjo, Xiaoxing Kou, Chider Chen, Dawei Liu, Yukari Watanabe, Chikako Hayashi, Hiroaki Yamato, Karen Yotsumoto, Urara Tanaka, Takaharu Taketomi, Takeshi Uchiumi, Anh D. Le, Songtao Shi, Fusanori Nishimura

**Affiliations:** a Department of Periodontology, Division of Oral Rehabilitation, Faculty of Dental Science, Kyushu University, Fukuoka, Japan; b Department of Anatomy and Cell Biology, University of Pennsylvania School of Dental Medicine, Philadelphia, PA, USA; c Department of Oral and Maxillofacial Surgery and Pharmacology, University of Pennsylvania School of Dental Medicine, PA, USA; d South China Center of Craniofacial Stem Cell Research, Guanghua School of Stomatology, Sun Yat-sen University, Guangdong, China; e Department of Orthodontics, Peking University School and Stomatology, Peking, China; f Dental and Oral Medical Center, Kurume University School of Medicine, Fukuoka, Japan; g Department of Clinical Chemistry and Laboratory Medicine, Graduate School of Medical Sciences, Kyushu University, Fukuoka, Japan

**Keywords:** Periodontal disease, Mesenchymal stem cell, Exosome, mirna, Osteoclastogenesis

## Abstract

Mesenchymal stem cell (MSC)–derived exosome plays a central role in the cell-free therapeutics involving MSCs and the contents can be customized under disease-associated microenvironments. However, optimal MSC-preconditioning to enhance its therapeutic potential is largely unknown. Here, we show that preconditioning of gingival tissue-derived MSCs (GMSCs) with tumor necrosis factor-alpha (TNF-*α*) is ideal for the treatment of periodontitis. TNF-*α* stimulation not only increased the amount of exosome secreted from GMSCs, but also enhanced the exosomal expression of CD73, thereby inducing anti-inflammatory M2 macrophage polarization. The effect of GMSC-derived exosomes on inflammatory bone loss were examined by ligature-induced periodontitis model in mice. Local injection of GMSC-derived exosomes significantly reduced periodontal bone resorption and the number of tartrate-resistant acid phosphatase (TRAP)-positive osteoclasts, and these effects were further enhanced by preconditioning of GMSCs with TNF-*α*. Thus, GMSC-derived exosomes also exhibited anti-osteoclastogenic activity. Receptor activator of NF-*κ*B ligand (RANKL) expression was regulated by Wnt5a in periodontal ligament cells (PDLCs), and exosomal miR-1260b was found to target Wnt5a-mediated RANKL pathway and inhibit its osteoclastogenic activity. These results indicate that significant ability of the TNF-*α*-preconditioned GMSC-derived exosomes to regulate inflammation and osteoclastogenesis paves the way for establishment of a therapeutic approach for periodontitis.

## 1. Introduction

Mesenchymal stem cells (MSCs) are multipotent progenitor cells that possess self-renewal with multiple differentiation capacities [1]. Besides its multipotency, immunoregulatory effects of MSCs have contributed to clinical investigations for many diseases involving tissue inflammation and autoimmune disorders [2]. Such striking observations of MSCs exerting functional improvement without engraftment or differentiation [3] have led to the development of a therapeutic approach that utilizes the secretome of MSCs [4]. MSCs secretes multiple trophic factors including cytokines, growth factors and exosomes, all of which serve as paracrine mediators for immunoregulation and tissue regeneration [5]. It has been well-established that soluble factors such as transforming growth factor-*β* (TGF-*β*), IL-10, prostaglandin E2 (PGE2), and indoleamine 2,3-dioxygenase (IDO) contribute to an MSC-mediated immunosuppression [6], while neutralizing these soluble factors does not completely abrogate the immunosuppressive activity of MSCs [7]. Meanwhile, emerging evidence have focused much attention to MSC-derived exosomes which play an important role in intercellular communication through their contents including cytokines, growth factors, mRNAs, and regulatory miRNAs with diverse combinations [8]. Specifically, exosomal miRNAs are capable of transferring genetic information [9], thereby inducing phenotypic changes in the recipient cells and enhancing the therapeutic effects of MSC-derived exosomes. MSCs regulates tissue homeostasis by controlling paracrine factors under inflammatory microenvironment [5]. Recent studies have indicated that appropriate preconditioning of MSCs with disease-related stimuli can optimize contents of exosomes to efficiently support the repair of specific diseases [10]. However, the proteins or miRNA expression profiles contained in exosomes are known to be influenced by the pre-treatment regimens. Therefore, the establishment of optical molecular-based protocol for MSC-preconditioning needs to be investigated.

Periodontitis is the most common osteolytic inflammatory disease in human that adversely affects systemic disorders, such as atherosclerosis, rheumatoid arthritis, and diabetes [11]. The pathology represents a typical osteoimmune disorder characterized by inflammation of the periodontium and subsequent destruction of the tooth-supporting tissue as alveolar bone that is a major cause of tooth loss in adults [12]. Accumulation of periodontal bacteria-associated biofilm initiates periodontitis, but is not fully sufficient to induce the disease as the host immune response is critical for the onset and progression [13]. Macrophages play an important role in the immune response both during the onset and resolution of inflammation in periodontal disease [14]. Macrophages are broadly classified into two phenotypes, pro-inflammatory M1 and wound-healing M2 cells, which correlate with Th1/Th2 nomenclature [15]. Classically activated M1 macrophages are induced by Th1 cytokines such as IFN-*γ* or lipopolysaccharide (LPS) and trigger acute inflammation by producing inflammatory cytokines such as IL-1*β*, TNF-*α*, and IL-6. In contrast, alternatively activated M2 macrophages are induced by IL-4 and IL-13 through activation of the common IL-4 receptor *α* signaling and produce anti-inflammatory cytokines such as IL-10, TGF-*β*, and VEGF [16].

Recently, adenosine signaling has been reported to influence the generation of macrophage polarization [17]. Adenosine is mainly produced by the CD39 and CD73 constitutively expressed on Treg cells, which coordinately produce adenosine from ATP to mediate immune suppression independent of IL-10 and TGF-*β* [18]. As a mediator of inflammation, ATP can be released from both host and bacterial cells under the circumstances of inflammation triggered by bacterial infection or tissue damage [19], while extracellular adenosine is a potent endogenous immunosuppressive mediator that is critical to the maintenance of homeostasis. In the presence of CD39 and CD73, ATP is hydrolyzed to adenosine and adenosine-A_2A_ receptor (R)-mediated - M1 macrophage activity [20, 21]. Furthermore, adenosine signaling via A_2B_ R converts phenotypic switch from M1 to M2 macrophages [22]. Under the periodontal infection, inflammatory mediators such as TNF-*α* and IL-17 are expressed by cells close to the alveolar bone and such mediators up-regulate the production of ligand for receptor activator of NF-*κ*B (RANKL), which plays essential role for osteoclast-mediated bone resorption [23]. Macrophages are involved in bone homeostasis and the increased M1/M2 ratio leads to enhanced osteoclastogenesis [24]. As M2 macrophages contribute to the tissue-remodeling process [25], their induction results in decreased inflammation and bone loss in periodontal tissues.

Compared to other somatic MSCs, dental tissue-derived MSCs possess the unique characteristics including clinical advantages of easy access in large quantities, as well as remarkable therapeutic potential by their secretome [26]. In particular, gingival tissue-derived MSCs (GMSCs) have a neural crest origin [27] and exhibit profound immunomodulatory and proliferative capacities with stable functional characteristics at higher passage numbers [28]. We previously reported that macrophages co-cultured with human GMSCs acquired M2 phenotype and systemically infused GMSCs promoted M2 macrophage infiltration to enhance wound healing [29]. Furthermore, we found that GMSCs secreted higher amounts of exosomes than those secreted by bone-marrow-derived MSCs (BMSCs) and TNF-*α* preconditioning enhanced release of IL-1RA-containing exosomes [30]. However, the effect of GMSC-derived exosomes on human osteoimmunological diseases is not known. Here, we examined the therapeutic effect of TNF-*α* preconditioned-GMSC-derived exosomes on periodontal disease, and tried to unveil detailed molecular mechanisms. TNF-*α*-enhanced exosomal CD73 expression contributed to M2 macrophage polarization and exosomal miR-1260b was critical regulator for suppressing inflammatory bone loss. Accordingly, our findings at the molecular level prove a therapeutic strategy for patients with periodontitis and other inflammatory osteoimmune disorders.

## 2. Materials and methods

### 2.1. Ethics statement and preparation of human tissue samples

All human gingival tissues were obtained as discarded clinical samples under the approved Institutional Review Board (IRB) protocol at the University of Pennsylvania and Kyushu University Hospital. The procedures for using human samples were conducted in accordance with the Declaration of Helsinki and approved by the Kyushu University Institutional Review Board for Human Genome/Gene Research (Protocol Number: 2019–374). Written informed consent was obtained from all subjects. All animal experimental protocols were approved by the Institutional Animal Care and Use Committee of Kyushu University and carried out in accordance with the approved guidelines.

### 2.2. Mice

C57BL/6NCrSlc (female, 8-week-old) were purchased from Japan SLC (Hamamatsu, Japan) and used under an institutionally approved animal research protocol (Kyushu University, Protocol #A19–284-0).

### 2.3. Cytokines and reagents

Recombinant human TNF-*α*, interferon-*γ* (IFN-*γ*), IL-4, IL-13, macrophage-colony stimulating factor (M-CSF), and Wnt5a were purchased from Biolegend (San Diego, CA, USA). LPS from *Escherichia coli* 055:B5 and acetylsalicylic acid were obtained from Sigma-Aldrich (St. Louis, MO, USA). JNK inhibitor SP600125 was purchased from Wako Pure Chemical Industries Ltd. (Osaka, Japan).

### 2.4. Cell culture

Human gingival tissue-derived MSCs (GMSCs) were isolated and cultured as reported in our previous studies [28–30]. Briefly, gingival tissues were gently separated, minced, and digested with a phosphate-buffered saline (PBS) solution containing collagenase type I (2 mg/mL) (Worthington Biochemicals, Lakewood, NJ, USA) and dispase II (4 mg/mL) (Sanko Junyaku, Tokyo, Japan) for 1 h at 37 °C. Single-cell suspensions from the gingiva were obtained by passing the culture through a 70-*μ*m strainer (Falcon, BD Biosciences Discovery Labware, Bedford, MA). All nucleated cells were seeded on 100-mm culture dishes with complete media containing Alpha modification of Eagle’s Medium (Invitrogen, Waltham, MA, USA) supplemented with 10% fetal bovine serum (FBS, Hyclone Laboratories, USA), 2 mM L-glutamine (Invitrogen, Carlsbad, CA, USA), 10 mM L-ascorbic acid phosphate (FUJIFILM Wako, Japan), 100 U/mL penicillin/streptomycin (Gibco Life Technologies, country), followed by an initial incubation for 48 h at 37 °C in 5% CO_2_ and 95% humidity. Cultures were subsequently washed with PBS twice to eliminate nonadherent cells. Attached cells were further cultured for another 12 days under the same conditions in the complete medium mentioned above and cells at the 3rd–5th passages were used for subsequent experiments. Human CD14^+^ peripheral blood-derived monocytes (PBMCs) were purchased from Lonza (Basel, Switzerland). Cells were seeded in 6-well plates (2 × 10^5^ cells/well) and cultured in RPMI 1640 medium (Nacalai Tesque, Kyoto, Japan) supplemented with 10% heat-inactivated FBS, 2 mM glutamine (Nacalai Tesque), 1% sodium pyruvate (Nacalai Tesque), 1% non-essential amino acids (Nacalai Tesque), and 25 ng/mL of M-CSF. To generate macrophages, monocytes were cultured for seven days with a single change of fresh medium at day 3 to day 4 after the initiation of the culture. To polarize into M1 or M2 macrophages, macrophages were further treated with 10 ng/mL of LPS plus 20 ng/mL of IFN-*γ* or 20 ng/mL of IL-4 plus 20 ng/mL of IL-13 for 24 h, respectively. Human periodontal ligament (PDL) cells (Lonza) were grown in a complete fibroblast medium using the FibroLife S2 Comp Kit (Kurabo Industries Ltd., Osaka, Japan), and cells at the 3rd–5th passages were used for subsequent experiments.

### 2.5. Characterization of GMSCs

Characterization of colony forming unit-fibroblasts (CFU-Fs) and multi-differentiation capacity of GMSCs were performed as per previously described methods [28, 31]. Isolated mononuclear cells were seeded at a density of 1 × 10^3^ per dish in 100-mm culture dishes containing the regular medium. Adherent cells were cultured for 18 days. The cultures were subsequently treated with PBS containing 0.1% Crystal violet and 4% paraformaldehyde (PFA). Cell clusters containing > 50 cells were counted as single colonies (CFU-F) under a Primovert microscope (Zeiss). GMSCs at the 3rd-5th passages were used for multi-differentiation capacity into adipocytes, osteoblasts, and chondrocytes. For adipogenic differentiation, GMSCs at 70%–80% confluence were cultured in an osteogenic medium containing *α*-MEM supplemented with 0.25 *μ*M dexamethasone, 0.5 mM 3-isobutyl-1-methylxanthine, 5 *μ*g/mL insulin (Cell Science & Technology Institute, Sendai, Japan), and 2 mM glutamine. For osteogenic differentiation, GMSCs at 70%–80% confluence were cultured in an osteogenic medium containing *α*-MEM supplemented with 2 mM *β*-glycerophosphate (Wako, Osaka, Japan) and 50 *μ*g/mL ascorbic acid (Wako). For chondrogenic differentiation, GMSCs (5 × 10^5^ cells) were cultured in MesenCult™ -ACF, a chondrogenic differentiation medium (STEMCELL™ Technologies, Canada), according to the manufacturer’s instructions.

### 2.6. Flow cytometry

The expression of CD73, CD90, CD105, CD34, CD45, and CD11b in GMSCs and CD11b, CD206, and CD86 in PBMC-derived macrophages were analyzed using the FACS Calibur (Becton Dickinson, USA) and the CellQuest software (Becton Dickinson). The adherent cells were washed with PBS and collected using Accutase (Nacalai Tesque) and resuspended in 50 *μ*L staining buffer (BD Pharmingen, country). The harvested cells were blocked with 2 *μ*L Human Trustain FcX (Fc receptor Blocking Solution; BioLegend) for 10 min at room temperature, and stained with 2 *μ*L antibodies namely, FITC anti-human CD73, FITC anti-human CD90, Alexa Fluor® 488 anti-human CD105, FITC anti-human CD34, PE anti-human CD45, FITC anti-human CD11b, Alexa Fluor® 488 anti-human CD11b, PE anti-human CD206, APC anti-human CD206, PE anti-human CD86 and APC anti-human CD86 (BioLegend) in the dark for 30 min at 4 °C.

### 2.7. Isolation and characterization of exosomes

Extracellular vesicles were prepared according to International Society of Extracellular Vesicles (ISEV) recommendations [32]. For preconditioning of GMSCs, cells in 100-mm culture dishes were grown to achieve a confluence of 70%–80%. After the medium was aspirated, cells were rinsed three times with PBS and treated with 100 ng/mL of recombinant human TNF-*α*, IFN-*γ*, LPS (FUJIFILM Wako), and acetylsalicylic acid (Sigma-Aldrich) in a serum-free medium or serum-free medium alone and incubated for 48 h prior to supernatant harvesting. GMSC-derived exosomes were isolated from the serum-free conditioned media using the MagCapture™ Exosome Isolation Kit PS (FUJIFILM Wako) [33]. Cells were preconditioned in a serum-free medium for 48 h. Medium was then collected, centrifuged at 10 000 × g for 30 min to eliminate other large extracellular vesicles (EV). Cleared supernatants were passed through 0.22 mm filter membranes and concentrated using a Vivaspin-20 concentrator (Sartorius, Göttingen, Germany). GMSC-derived exosomes were also isolated by differential centrifugation, as previously described [30], to compare the characteristics of exosomes between the two isolation procedure (Fig. S1). Amounts of protein in exosome were quantified using the BCA Protein Assay Kit (Takara Bio, Otsu, Japan). Transmission electron microscopy (TEM) analyses were performed by the Hanaichi Ultrastructure Research Institute (Okazaki, Japan) as previously reported [34]. The Nanoparticle Characterization System (NanoSight, Malvern Instruments, UK) was used to measure size distribution and concentration of exosomes. For treating exosomes with CD73 neutralizing antibodies, 10 *μ*g of exosomes was incubated with 1 *μ*g of rabbit anti-CD73 antibody (N1N3, GeneTex, Irvine, CA) for 1 h at room temperature, and unreacted antibodies were removed using ExoQuick-TC reagent (SBI, Palo Alto, USA) according to the manufacture’s instruction. Anti-normal rabbit (#2729, Cell Signaling Technology) was used for negative control.

### 2.8. Cellular uptake of exosomes

GMSC-derived exosomes were labeled using an ExoSparkler Exosome Membrane Labeling Kit-Green (Dojindo, Kumamoto, Japan) according to the manufacturer’s protocols. Then, the green fluorescence-labeled exosomes were added to PBMC-differentiated macrophages at a final concentration of 1 *μ*g/mL and incubated for 3 h. Following incubation, cells were washed with PBS (pH 7.4), fixed with 4% PFA. Non-specific reactions were blocked with 3% BSA in PBS. Macrophages were labelled with Alexa Fluor 594 phalloidin (Thermo Fisher Scientific) for F-actin, or PlasMem Bright Red (Dojindo) for cell membrane and 4′,6-diamidino-2-phenylindole (DAPI) (Thermo Fisher Scientific) was used for nucleus. For Coverslips were mounted using the PermaFluor Mounting medium (Thermo Fisher Scientific) and images were analyzed by LSM 700 confocal microscope (Carl Zeiss) and Zen 2012 software.

### 2.9. Wound healing model in mice

Mice were randomly divided into the following three groups (*n* = 5): (1) the Placebo (PBS) group, (2) the GMSC-derived exosomes group (Exo-Ctrl), and (3) the TNF-*α*-preconditioned GMSC-derived exosomes group (Exo-TNF). The excisional full-thickness skin wound splinting model was generated in accordance with our previous study [30]. Briefly, full-thickness square excision wounds (10 × 10 mm^2^) were created by marking the wound area on the mid-backs of the mice. The cutaneous wounds were subcutaneously injected with either placebo or GMSC-derived exosomes (200 *μ*g) dissolved in PBS (200 *μ*L). A series of digital photographs of the cutaneous wounds was taken using a standard ruler as scale. At the indicated time points, percentage of wound closure was quantified on photographs using the Adobe Photoshop Elements 14 software (Adobe Systems, USA). Changes in the area of the cutaneous wounds were expressed as a percentage of the initial wound area.

### 2.10. Histology and immunohistochemistry

Standard hematoxylin and eosin (H&E) staining and dual-color immunofluorescence analysis using specific primary antibodies for mice F4/80 (1:100, MCA497GA, Bio-Rad Laboratories, Inc., Hercules, CA, USA) and arginase (1:1000, D4E3M, Cell Signaling Technology, Danvers, MA, USA) were performed as per previously described methods [29].

### 2.11. Ligature-induced periodontal model in mice

Mice were randomly divided into the following four groups (*n* = 5): (1) Unligated + Placebo (PBS) group, (2) Ligated + Placebo (PBS) group, (3) Ligated + GMSC-derived exosomes (Exo-Ctrl) group, and (4) the TNF-*α*-preconditioned GMSC-derived exosomes (Exo-TNF) group. To induce periodontal bone loss in mice, a 5–0 silk ligature (Akiyama Medical MFG. Co., Tokyo, Japan) was tied around the left maxillary second molar, in accordance with Abe et al. [35]. After ligation, either placebo or exosomes (20 *μ*g) suspended in PBS (20 *μ*L) were injected into the palatal gingiva of the ligated second maxillary molar using a 33-gauge needle Hamilton syringe (Hamilton Company, NV, USA). The contralateral molar tooth in each mouse was left unligated (baseline control for bone loss measurements). The ligations remained intact in all mice throughout the experimental period. After seven days, mice were euthanized, and the maxillae were removed for further analysis. To evaluate the alveolar bone loss, the maxillae were treated with 0.5% sodium hypochlorite solution for three days, and 3% hydrogen peroxide for one day. After washing with PBS, the maxillae were stained with 0.05% Toluidine blue. Periodontal bone loss in defleshed maxillae was assessed morphometrically using an Olympus DP72 digital camera. Specifically, alveolar bone loss was measured from the cemento-enamel junction (CEJ) of the third mesial root to the pinnacle of the alveolar bone (AB), and from the distal and mesial roots of the second and first molars. The alveolar bone loss of each group was defined as the sum of distances from the five sites based on previous study [35]. The area of palatal alveolar bone loss around the maxillary secondary molars was pictured and measured using the Olympus DP-11BSW software (Olympus, Japan) according to the previous study [36]. Schematic illustration of these measurements were included in Fig. S2.

### 2.12. Tartrate-resistant acid phosphatase (TRAP) staining and osteoclast quantification

Maxillae were fixed in a 4% paraformaldehyde solution for 24 h and decalcified in 10% EDTA solution at 4 °C for four weeks. The decalcified specimens were dehydrated, embedded in paraffin, and sectioned at a thickness of 5.0 *μ*m. A part of each tissue section was subjected to tartrate-resistant acid phosphatase (TRAP) staining using the TRAP/ALP Stain Kit (FUJIFILM Wako). Multinucleated TRAP-positive cells formed on the surface of alveolar bone around the second molar were counted as active osteoclasts. Photographs were taken using BZ8000 (Keyence Co., Osaka, Japan) and the number of TRAP-positive cells were counted.

### 2.13. Quantitative RT-PCR analysis

Total RNA was isolated from the cells using ISOGEN II (Nippon Gene, Tokyo, Japan), and first-strand cDNA was synthesized using the PrimeScript RT Master Mix (Takara Bio, Otsu, Japan). qRT-PCR was carried out using the KAPA SYBR® FAST qPCR kit Master Mix (Kapa Biosystems, Woburn, MA, USA) on a StepOnePlus ™ Real-Time System (Applied Biosystems, Carlsbad, CA, USA) with the following conditions: 95 °C for 3 min, 40 cycles of 95 °C for 3 s, and 60 °C for 30 s. To correct varying copies of first-strand cDNA templates, a passive reference dye (Rox) was added to the PCR master mix. The results were recorded and analyzed using the StepOne™ software V2.2.2 (Applied Biosystems, Life Technologies Corporation) utilizing the auto calculated threshold cycle. The ΔΔC_T_ method was performed to calculate individual gene’s relative expression levels and glyceraldehyde-3-phosphate dehydrogenase (*GAPDH*) was used as an internal control. The primer sequences used in this study are listed in Table S1.

### 2.14. Western blot analysis

Cells were washed with PBS and lysed in the Cytobuster Protein Extraction Reagent (Novagen Inc., Madison, Wisconsin, USA) supplemented with a protease inhibitor cocktail (Nacalai Tesque). Protein samples were separated on polyacrylamide gels and then transferred onto polyvinylidene difluoride membranes. The membranes were incubated with the appropriate primary antibodies: anti-CD9 (1:1000, Ts9, Thermo Fisher Scientific), anti-NT5E/CD73 (1:1000, D7F9A, Cell Signaling Technology), anti-CD63 (1:1000, Ts63, Thermo Fisher Scientific), anti-CD81 (1:1000, Ts81, Thermo Fisher Scientific), anti-Calnexin (1:1000, C5C9, Cell Signaling Technology), anti-RANKL (1:1000, 12A668, Novus Biologicals, USA), anti-TNFRSF11B (1:1000, 5G2, Invitrogen), anti-Wnt5a (1:1000, C27E8, Gene Tex, USA), anti-p44/42 MAPK (ERK1/2) (1:1000, 137F5, Cell Signaling Technology), anti-Phospho-p44/42 MAPK (Erk1/2) (Thr202/Tyr204) (1:1000, D13.14.4E, Cell Signaling Technology), anti-pan-Akt (1:1000, C67E7, Cell Signaling Technology), anti-Phospho-Akt (Ser473) (1:1000, D9E, Cell Signaling Technology), anti-JNK (1:1000, 56G8, Cell Signaling Technology), anti-Phospho-JNK (Thr183/Tyr185) (1:1000, 81E11, Cell Signaling Technology), and anti-*β*-actin (1:1000, 13E5, Cell Signaling Technology). The blotted membranes were visualized by using a densitometry technique on a LAS-4000 mini luminescent image analyzer (GE Healthcare Marlborough, USA) and quantified using the Multi Gauge 3.1 software (FUJIFILM, Tokyo, Japan).

### 2.15. Microarray analysis of exosomal miRNA

To identify exosomal miRNA in GMSCs preconditioned and induced by TNF-*α*, comprehensive miRNA-expression profiles of exosomes were analyzed in culture media from three patient-derived GMSCs with or without TNF-*α*-treatment including the 2565 probes for human mRNAs, using the 3D-Gene® Human miRNA Oligo Chip ver.21 (TORAY, Kanagawa, Japan). Total RNA was extracted from the exosomes using the 3D-Gene® RNA extraction reagent and a liquid sample kit (TORAY, Kanagawa, Japan). Comprehensive miRNA expression analysis was performed using a 3D-Gene® miRNA Labeling kit (TORAY, Kanagawa, Japan) and a 3D-Gene® Human miRNA Oligo Chip (TORAY, Kanagawa, Japan). The annotation and oligonucleotide sequences of the probes were confirmed using the miRBase (http://microrna.sanger.ac.uk/sequences/). After repeated cycles of stringent washes, fluorescence signals were scanned using the 3D-Gene Scanner (Toray Industries Inc., Tokyo, Japan) and analyzed using the 3D-Gene Extraction software (Toray Industries Inc.). The relative expression level of a given miRNA was calculated by comparing the signal intensities of valid spots for all microarray experiments.

### 2.16. Cell transfection

Transfection of siRNA and miRNA mimics were carried out using the Lipofectamine™ RNAiMAX Transfection Reagent (Thermo Fisher Scientific) for 24 h according to our reverse transfection protocol [37]. For knockdown analysis, Stealth™ RNAi duplexes against human WNT5A, a mixture of three different siRNAs (HSS11355, HSS11356, and HSS187692), were obtained from the Invitrogen Corporation (Invitrogen Life Technologies, Carlsbad, CA). Stealth™ RNAi negative control duplex (Medium GC Duplex, Invitrogen Life Technologies) was used as a control. For miRNA mimic transfection, PDL cells were transfected with 20 nM miRCURY LNA microRNA Mimic for hsa-miR-1260b, hsa-miR-4454, and hsa-miR-5100 (Qiagen, Germany) or control miRNA (negative control: celmiR-39–3p, Qiagen).

### 2.17. Statistical analysis

Data were analyzed using the JMP Pro 14 software (SAS Institute Inc, Cary NC, USA). Comparisons between two groups were performed using independent unpaired two-tailed Student’s *t-*test, and comparisons between more than two groups were performed using one-way analysis of variance (ANOVA) with Bonferroni correction. *P* values of < 0.05 were considered statistically significant.

## 3. Results

### 3.1. Identification of GMSCs

**Fig. 1.**
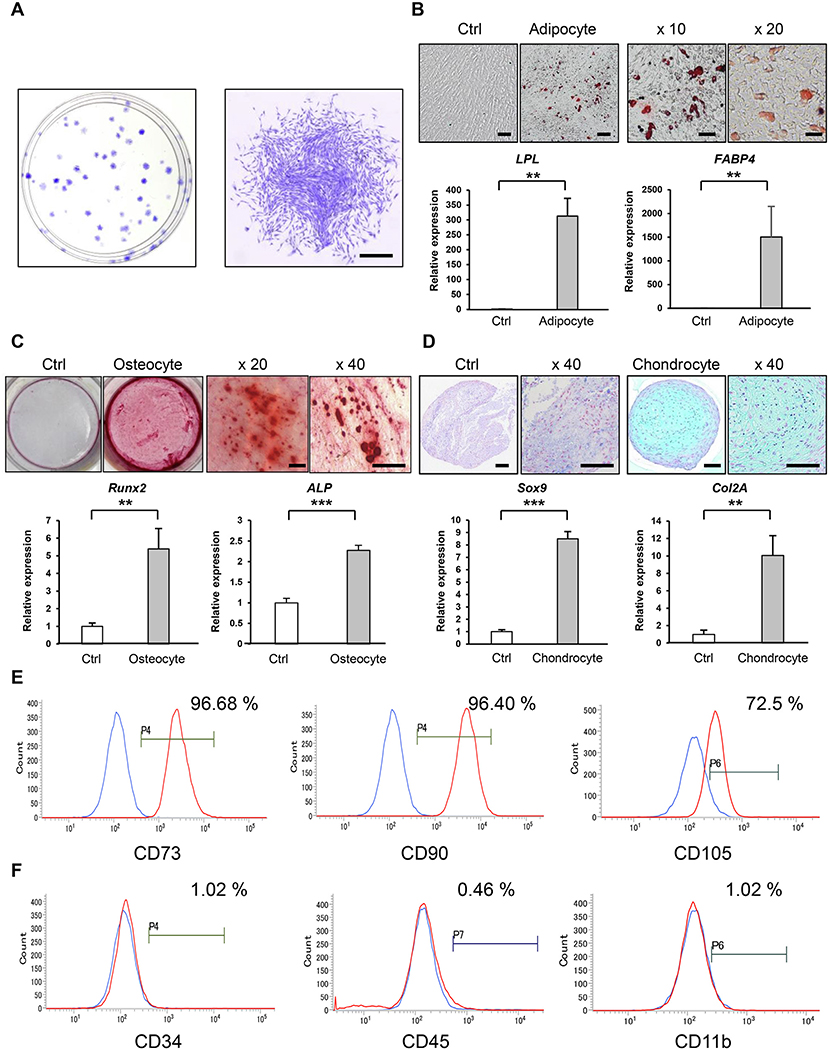
Characterization of stem cells isolated from human gingival tissues **(A)** Capability of CFU-Fs of GMSCs. Toluidine blue staining. Representative images of CFU-Fs on a culture dish (**left**) and fibroblastic colony-forming cells (**right**). **(B)** Adipogenic differentiation of GMSCs. Representative images of Oil Red O staining and adipocyte-specific gene expression (LPL and FABP4) were analyzed using qRT-PCR. **(C)** Osteogenic differentiation of GMSCs. Representative images of Alizarin Red staining and osteogenesis-specific genes expression (Runx2 and ALP) were analyzed using qRT-PCR. **(D)** Chondrogenic differentiation of GMSCs. Representative images of Alcian blue staining and chondrogenic differentiation-related gene expression (Sox9 and Col2A) were analyzed using qRT-PCR. **(E) and (F)** Immunophenotype of GMSCs. Surface expression of MSC-positive **(E)** and negative **(F)** markers on GMSCs were evaluated using flow cytometry. Blue histograms: GMSCs stained with control antibody; Red histograms: GMSCs stained with antibodies against cell surface antigens. Percentiles indicate the average of each antigen. **p < 0.01; ***p < 0.001. Error bars are means ± SD. Data were analyzed using independent unpaired two-tailed Student’s *t*-test. Scale bar = 500 *μ*m. (For interpretation of the references to color in this figure legend, the reader is referred to the web version of this article.)

Firstly, we isolated MSCs from human gingival tissues using the CFU-F approach [28], which is a classical gold standard method [38]. GMSCs were independently attached to the plastic culture dishes and to form CFU-Fs, which demonstrated that GMSCs exhibited greater clonal expansion and differentiation (Fig. 1A). Multipotency of GMSCs were assessed under induced adipogenic, osteogenic, and chondrogenic conditions, and determined using Alizarin Red S (Fig. 1B), Oil Red O (Fig. 1C) and Alucian blue (Fig. 1D) staining methods, respectively. Adipogenic differentiation was further confirmed using qRT-PCR to detect the increased expression of adipocyte-specific genes including lipoprotein lipase (LPL) and fatty acid binding protein (FABP4) (Fig. 1B). Similarly, induction of osteocyte- and chondrocyte-specific genes was confirmed using runt-related transcription factor 2 (Runx2) and alkaline phosphatase (ALP) (Fig. 1C), SRY-Box transcription factor 9 (Sox9) and type II collagen (Col2A) (Fig. 1D), respectively. Flow cytometric analysis demonstrated that GMSCs were positive for CD73, CD90, and CD105 (Fig. 1E), but negative for hematopoietic cell markers such as CD34, CD45, and CD11b (Fig. 1F). These data revealed that the isolated GMSCs possessed exhibit identical characteristics for MSCs.

### 3.2. Exosomes from TNF-*α*-stimulated GMSCs enhance M2 macrophage polarization

**Fig. 2.**
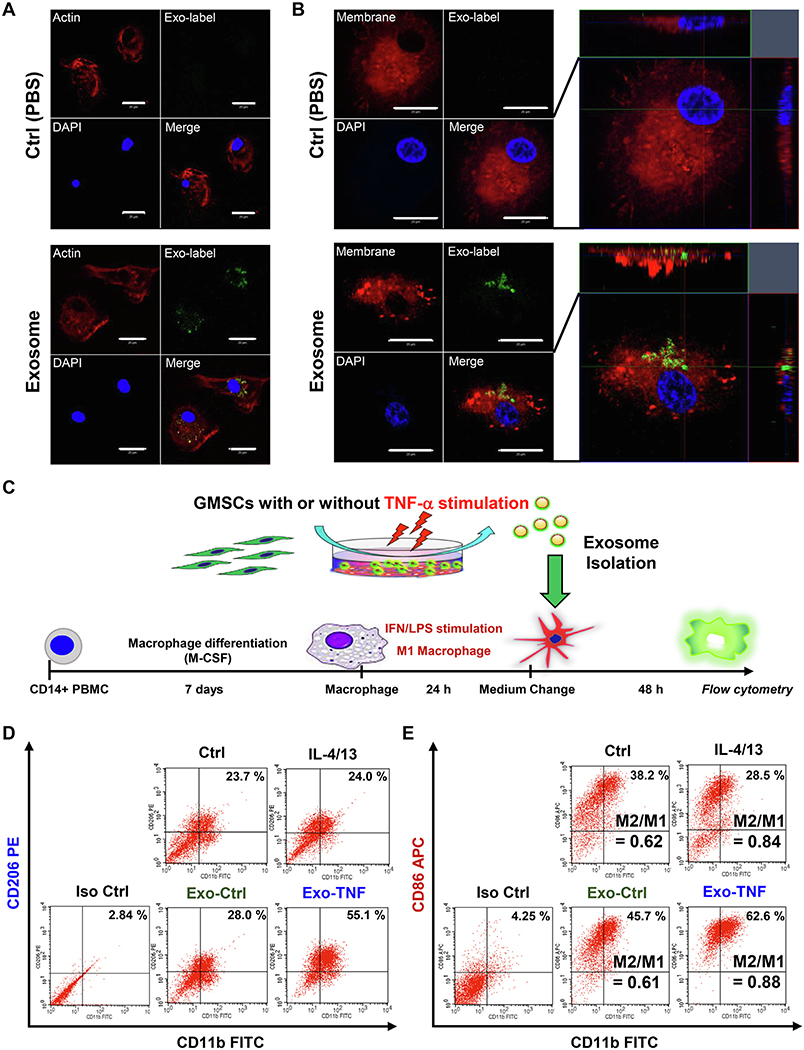
Effect of TNF-*α*-treated GMSC-derived exosomes on macrophage polarization **(A) and (B)** Cellular uptake of GMSC-derived exosomes into human macrophages differentiated from PBMC. ExoSparker™-labeled exosomes (green) were incubated with macrophages for 3 h. Macrophages were labelled with F-actin (red) or plasma membrane (Red) and DAPI for nuclei (blue). Scale bar = 20 *μ*m. **(B)** Confocal cross-section and z-stack images of macrophage demonstrates internalization of exosomes. **(C)** Strategy diagram for the validation of phenotypic switching capacity of exosomes from M1 to M2 macrophages. After M1 macrophages were generated by LPS and IFN-*γ* stimulation from resting macrophages derived from PBMC, exosomes from GMSC with or without TNF-*α* stimulation were incubated with M1 macrophages for 48 h. **(D)** Effects of TNF-*α*-treated GMSC-derived exosomes on CD206 expression in M1 macrophages. Percentage of double positive cells (CD11b^+^ CD206^+^) was analyzed to measure the ratio of macrophages polarized to M2 phenotype. **(E)** Effects of TNF-*α* treated GMSC-derived exosomes on CD86 expression in M1 macrophages. Percentage of double positive cells (CD11b^+^ CD86^+^ ) representing M1 macrophages was analyzed, and M2/M1 balance was determined by using the ratio of (CD11b^+^ CD206^+^)/(CD11b^+^ CD86^+^) macrophage population. (For interpretation of the references to color in this figure legend, the reader is referred to the web version of this article.)

To assess if the immunomodulatory capacity of GMSCs could be mediated by GMSC-derived exosomes into macrophages, we monitored cellular internalization of exosomes by macrophages. Taking the clinical translational value into consideration, we used human PBMC-differentiated macrophages. Fluorescently labeled exosomes from GMSCs were successfully internalized into macrophages in 3 h after the addition, as visualized by using confocal microscopy (Fig. 2A and B). To examine the biological effect of GMSC-derived exosomes on anti-inflammatory M2 macrophage polarization, macrophages were stimulated with exosomes for 48 h, and the expression of CD206, a well-accepted M2 marker, was validated by using flow cytometry (Fig. S3A). To further examine whether preconditioning of GMSCs with diverse stimuli could induce significant synergistic effect, GMSCs were stimulated either with LPS, TNF-*α*, IFN-*γ*, or acetylsalicylic acid (ASA) before isolation of exosomes from cell culture supernatant. To this end, standard activation of M1 and M2 was established by the addition of LPS and IFN-*γ* and IL-4 with or without IL-13, respectively. CD206^+^ macrophage population was up-regulated by M2 activation, while decreased by M1 activation. Stimulation with GMSC-derived exosomes alone enhanced M2 macrophage polarization, as compared to undifferentiated macrophage (59.3% vs. 64.4%) (Fig. S3B). Of all the remaining preconditions, TNF-*α*-stimulated GMSC-derived exosomes significantly enhanced M2 activation (68.9%) (Fig. S3B). M2/M1-related cytokine expression after stimulation with exosomes in resting macrophages was also determined by using qRT-PCR (Fig. S3C). Exosomes with or without preconditioning with TNF-*α* increased the expression of IL-10 at the same levels of IL-4/13 stimulation, while inflammatory M1-related IL-1*β*, TNF-*α*, and iNOS expressions were decreased. We further examined whether TNF-*α* preconditioned-GMSC-derived exosomes could shift macrophage marker expression from those of M1 to the M2 phenotype. To this end, M1-activated macrophages were stimulated with exosomes with or without TNF-*α* preconditioning, and the expression of M1 (CD86) and M2 marker (CD206) was examined (Fig. 2C). TNF-*α*-preconditioned GMSC-derived exosomes dramatically up-regulated CD206 expression (Fig. 2D), and the M2/M1 ratio was almost equal to IL4/13 stimulation (0.88 vs. 0.84) (Fig. 2 E). Taken together, these results suggest that TNF-*α* preconditioning enables GMSCs to secrete exosomes capable of converting macrophage phenotype from proinflammatory M1 to an anti-inflammatory M2.

### 3.3. Exosome from TNF-*α*-stimulated GMSCs facilitate skin wound healing

**Fig. 3.**
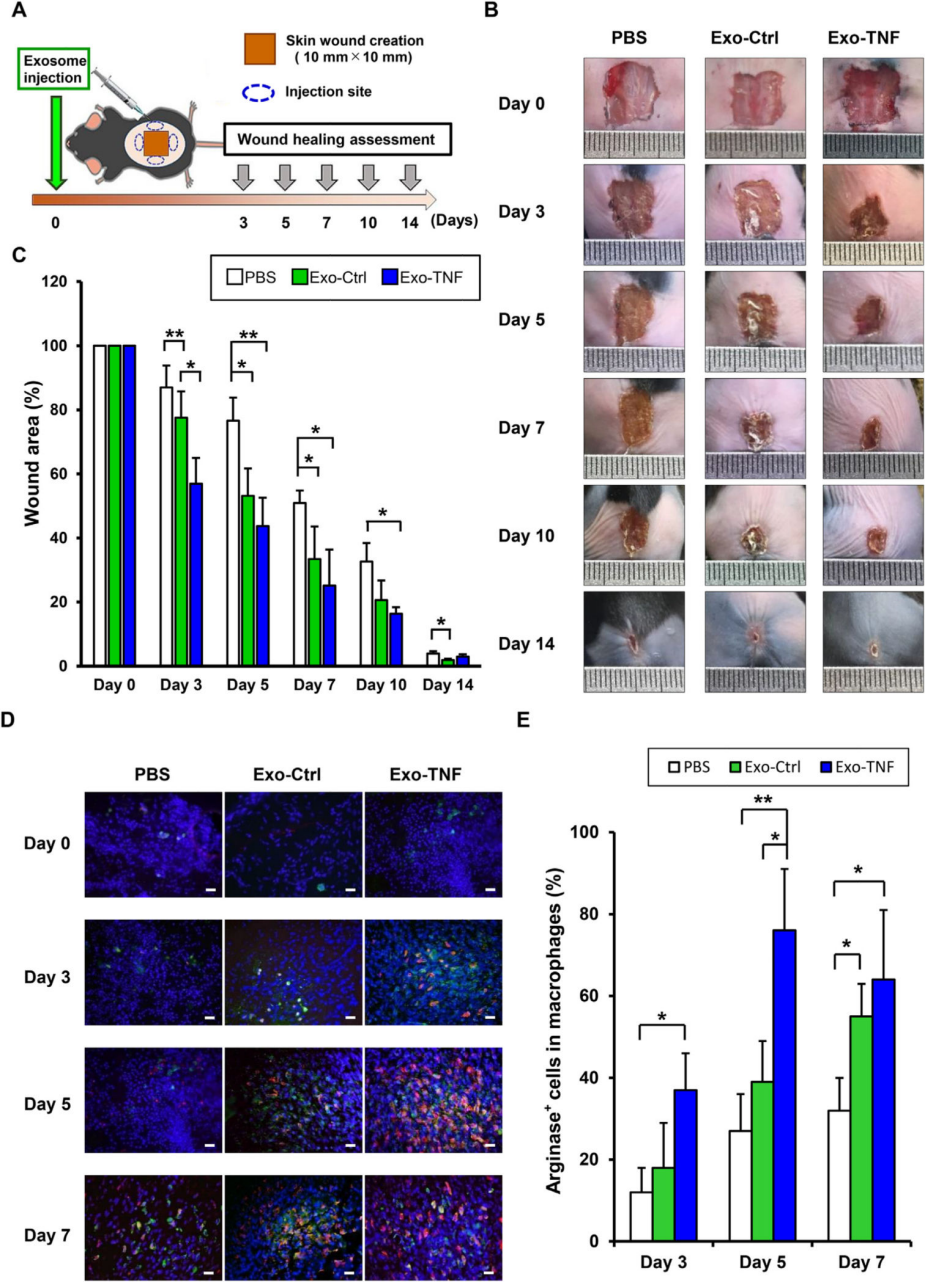
Therapeutic effect of GMSC-derived exosomes on skin wound healing in mice **(A)** Schematic illustration for skin wound healing mouse model and local administration of exosomes. **(B)** Representative healing process of cutaneous wounds in each group. A full-thickness 10 mm^2^ wound was made in C57BL/6 mice. Either placebo (PBS) or GMSC-derived exosomes (Exo-Ctrl), or TNF-*α*-preconditioned GMSC-derived exosomes (Exo-TNF) (200 *μ*g) dissolved in PBS (200 *μ*L) were injected subcutaneously as illustrated. **(C)** Wound closure kinetics (*n* = 5). The percentage of wound area was calculated as: (area of original wound – area of measured wound)/area of original wound × 100. **(D)** M2 macrophage detection in each wound. Frozen sections of full-thickness incisional skin wounds from mice after treatment with or without GMSC-derived exosomes for different days were immune-stained with DAPI (blue), F4/80 (green), and arginase-1 (red). Scale bar = 50 *μ*m. **(E)** Quantification of the percentage arginase + cells in F4/80 + cells according to immunofluorescence analysis. *p < 0.05, **p < 0.01. Error bars represent means ± SD. Data were analyzed using independent unpaired two-tailed Student’s *t*-tests. (For interpretation of the references to color in this figure legend, the reader is referred to the web version of this article.)

To gain further insight into the impact of TNF-*α*-preconditioned GMSC-derived exosomes (Exo-TNF) on M2 macrophage polarization, *in vivo* therapeutic effects of exosomes on wound healing were evaluated in a mice model (Fig. 3A). Compared to control mice (PBS), injection of GMSC-derived exosomes without preconditioning (Exo-Ctrl) displayed promoted skin wound healing at days 5 and 7 after wound creation. Moreover, Exo-TNF treatment revealed significantly augmented wound closure compared to Exo-Ctrl-treated mice, wherein the accelerated wound closure was observed as early as day 3 after wound creation (Fig. 3B and C). These macroscopic findings were confirmed using histological assessment (Fig. S4). Exo-TNF-injected wounds demonstrated fastest re-epithelialization compared to PBS and Exo-Ctrl-treated groups. At day 7, infiltration of inflammatory cells remained potent in the control group, while these cells were apparently decreased in exosome-treated wounds by day 7 (Exo-Ctrl) and day 5 (Exo-TNF), respectively. To further assess the exosome-mediated M2 macrophage induction in the wounds, tissues were stained for the macrophage marker F4/80 and arginase-1, another well-known marker for M2 macrophage [39] (Fig. 3D and 3E). Exo-Ctrl-treated wounds increased the expression of F4/80 and arginase-1-positive macrophages in a time-dependent manner. Furthermore, induction of arginase-1-positive macrophages reached the peak at day 5 post wounding in Exo-TNF-treated wounds, and the percentage of arginase-1-positive macrophages were significantly higher than Exo-Ctrl-treated wounds. These results demonstrated that GMSC-derived exosomes were capable of enhancing wound healing capacity and resolving inflammation by inducing M2 macrophage polarization, and TNF-*α* preconditioning enabled GMSCs to augment these effects.

### 3.4. TNF-*α*-stimulation increases CD73 expression in GMSC-secreted exosomes

**Fig. 4.**
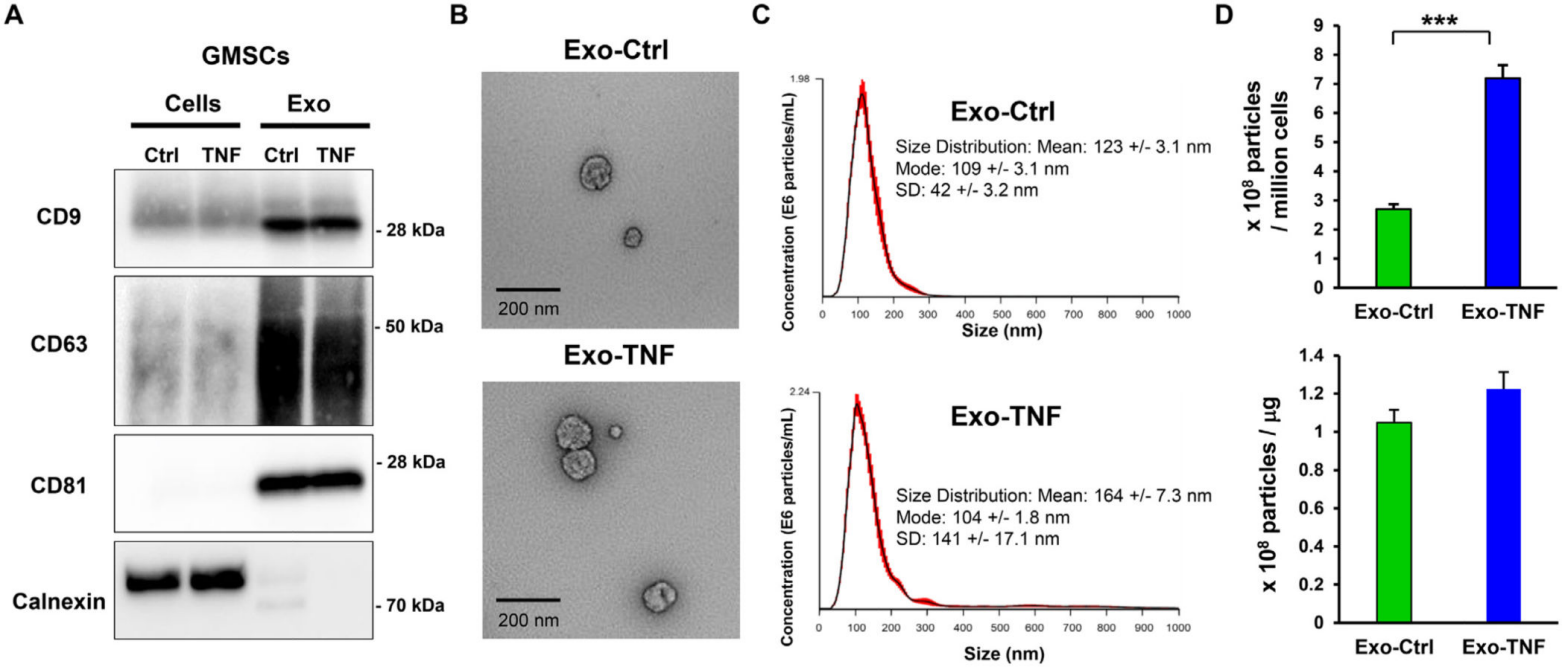
Identification of TNF-*α*-treated GMSC-derived exosomes **(A)** Western blot analysis showing the expression of CD9, CD63, CD81and Calnexin in GMSCs (Cells) and GMSC-derived exosomes (Exo). Protein samples from GMSCs and GMSC-derived exosomes with or without TNF-*α* treatment were subjected to analyses. Equal amounts of protein (5 *μ*g per lane) were loaded. **(B)** Representative transmission electron micrographs (TEM) of exosomes from Exo-Ctrl and Exo-TNF. Scale bar = 200 nm. **(C)** Particle size distribution analysis of Exo-Ctrl (**left**) and Exo-TNF (**right**) using the NanoSight analyzer. **(D, E)** Exosome particle production per 10^8^ cells **(D)** and exosome particle density per 1 *μ*g of the exosomal protein **(E)** were compared between Exo-Ctrl and Exo-TNF.

**Fig. 5.**
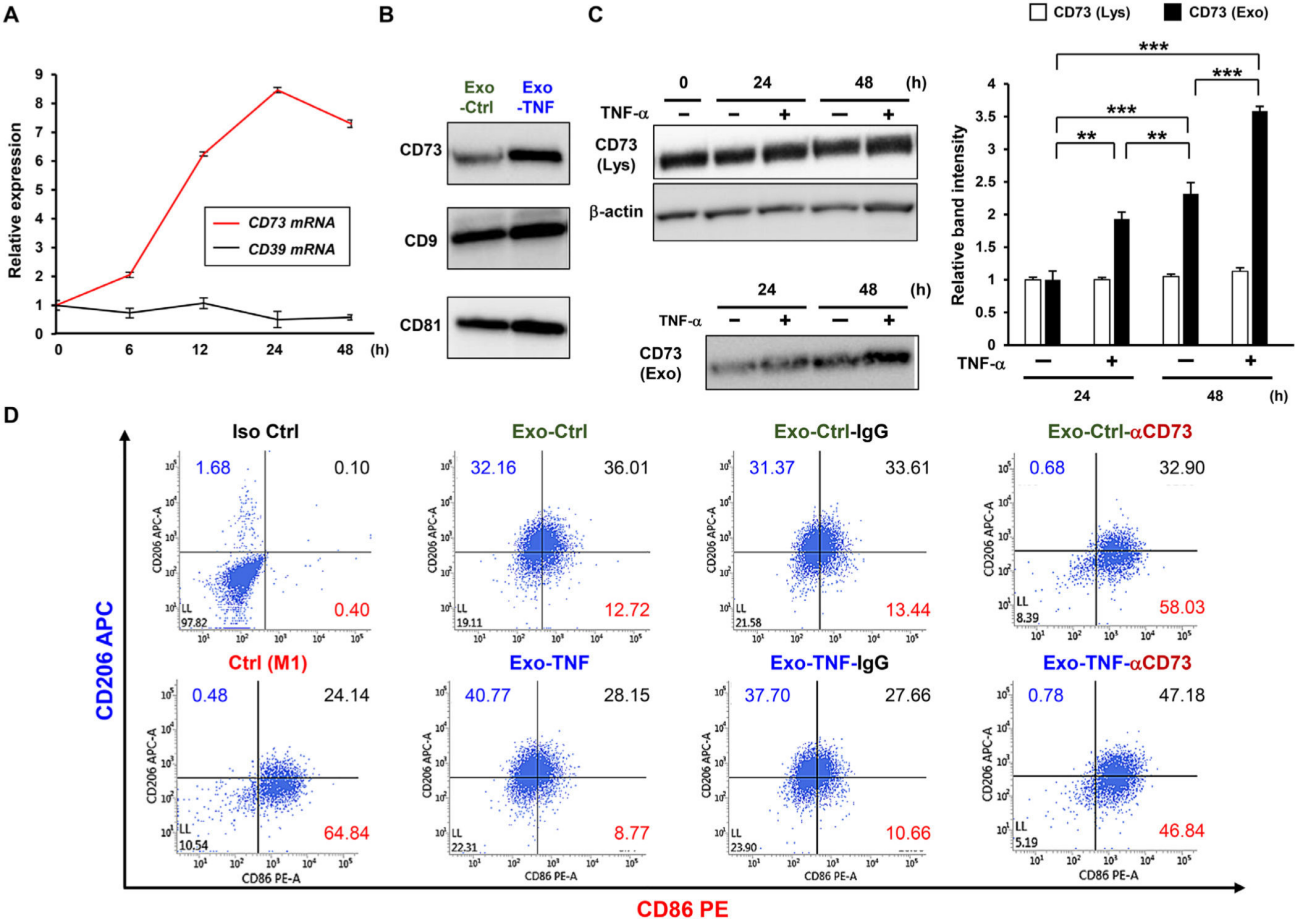
Effect of TNF-*α*-induced expression of exosomal CD73 on M2 macrophage polarization. **(A)** Time-course expressions of CD39 and CD73 mRNA in TNF-*α*-stimulated GMSCs. *GAPDH* was used as an internal control. **(B)** Exosomal CD73 protein expression in equal amounts of the protein (5 *μ*g) from Exo-Ctrl and Exo-TNF were compared. Exosome-associated CD9 and CD81 proteins were used as a loading control. **(C)** Western blot analysis indicating time-course expression of CD73 in GMSCs (Lys, **upper**) or GMSC-derived exosomes (Exo, **lower**) with or without TNF-*α* preconditioning. *β*-actin was used as a loading control for cytosolic protein. Relative CD73 protein expressions in GMSC (Lys; white bar) and GMSC-derived exosomes (Exo; black bar) were measured by quantifying the density of bands using the MultiGauge software. **(D)** Effect of CD73 neutralizing antibody on GMSC-derived exosome-induced M2 macrophage polarization. After M1 macrophages were generated by LPS and IFN-*γ* stimulation from PBMC, 10 *μ*g of CD73 neutralizing antibody (*α*CD73)-pretreated Exo-Ctrl and Exo-TNF were incubated with CD11b-gated M1 macrophage for 48 h. The population of CD206^+^ CD86^−^ cells in CD11b^+^ macrophages was counted for M2 macrophage polarization. Anti-rabbit IgG was used as control. **p < 0.01; ***p < 0.001. Error bars represent means ± SD., *n* = 3. Statistical analyses were performed using one-way analysis of variance (ANOVA) with Bonferroni correction.

Next, we explored the molecular mechanisms of TNF-*α* preconditioning on the secretion of exosomes from GMSCs inducing M2 macrophage polarization. Western blot demonstrated that TNF-*α* stimulation increased the secretion of exosome from GMSCs without affecting the expression of exosomal positive markers (CD9, CD63 and CD81), and both exosomes were free of cellular calnexin contaminants (Fig. 4A). The morphology of exosomes was compared using a transmission electron microscope (TEM). Both exosomes had the appearance of 100-nm saucer-like shape of typical exosomes [8] (Fig. 4B). Nanosight particle tracking analysis further confirmed the purity and size distribution of the exosomes (Fig. 4C). The most abundant particle sizes of Exo-Ctrl and Exo-TNF were almost the same (Mode: 109 ± 3.1 nm vs. 104 ± 1.8 nm). To validate the effect of TNF-*α*-preconditioning on exosome release, the number of GMSC-secreted Exo-Ctrl and Exo-TNF particles were normalized to those of manipulated cells or protein concentration. TNF-*α* stimulation significantly increased exosome release by approximately 2.7-fold (Fig. 4D), while it exerted little effect on exosomal protein density (Fig. 4E). In accordance with our established protocol, time course expressions of CD73 and CD39 mRNAs during TNF-*α*-preconditioning were monitored for 48 h. qRT-PCR analysis demonstrated that CD73 mRNA was significantly elevated within 12 h and kept constitutively higher for up to 48 h, although the expression level of CD39 was unchanged (Fig. 5A). Similarly, a significantly increased CD73 expression of Exo-TNF in comparison with Exo-Ctrl was observed (Fig. 5B). However, protein expression profiles of cellular CD73 in GMSCs were unchanged during TNF-*α*-preconditioning (Fig. 5C), thereby indicating that TNF-*α*-induced CD73 proteins were released by CD73-overexpressed Exo-TNF of GMSCs. We further explored whether exosomal CD73 expression is responsible for M2 macrophage polarization by using CD73 neutralizing antibody (Fig. 5D). In PBMC-differentiated CD11b+ M1 polarized macrophage, the percentage of M1 macrophage (CD86^+^ CD206^−^ cells) and M2 macrophage (CD86^−^CD206^+^ cells) were 64.86% and 0.48%. As expected, treatment of Exo-Ctrl and Exo-TNF converted macrophage phenotype from M1 to M2 (12.72% vs. 32.16% and 8.77% vs. 40.77%), respectively. While pretreatment of exosomes with IgG had little effect on these M1/M2 marker expressions, neutralizing anti-CD73 Ab-treated exosomes significantly abolished the induction of M1 to M2 macrophage polarization in Exo-Ctrl (58.03% vs. 0.68%) and Exo-TNF (46.84% vs. 0.78%). These result demonstrated that TNF-enhanced exosomal CD73 expression is essential for inducing M2 macrophage polarization.

### 3.5. GMSC-secreted exosomes inhibit periodontal bone loss

**Fig. 6.**
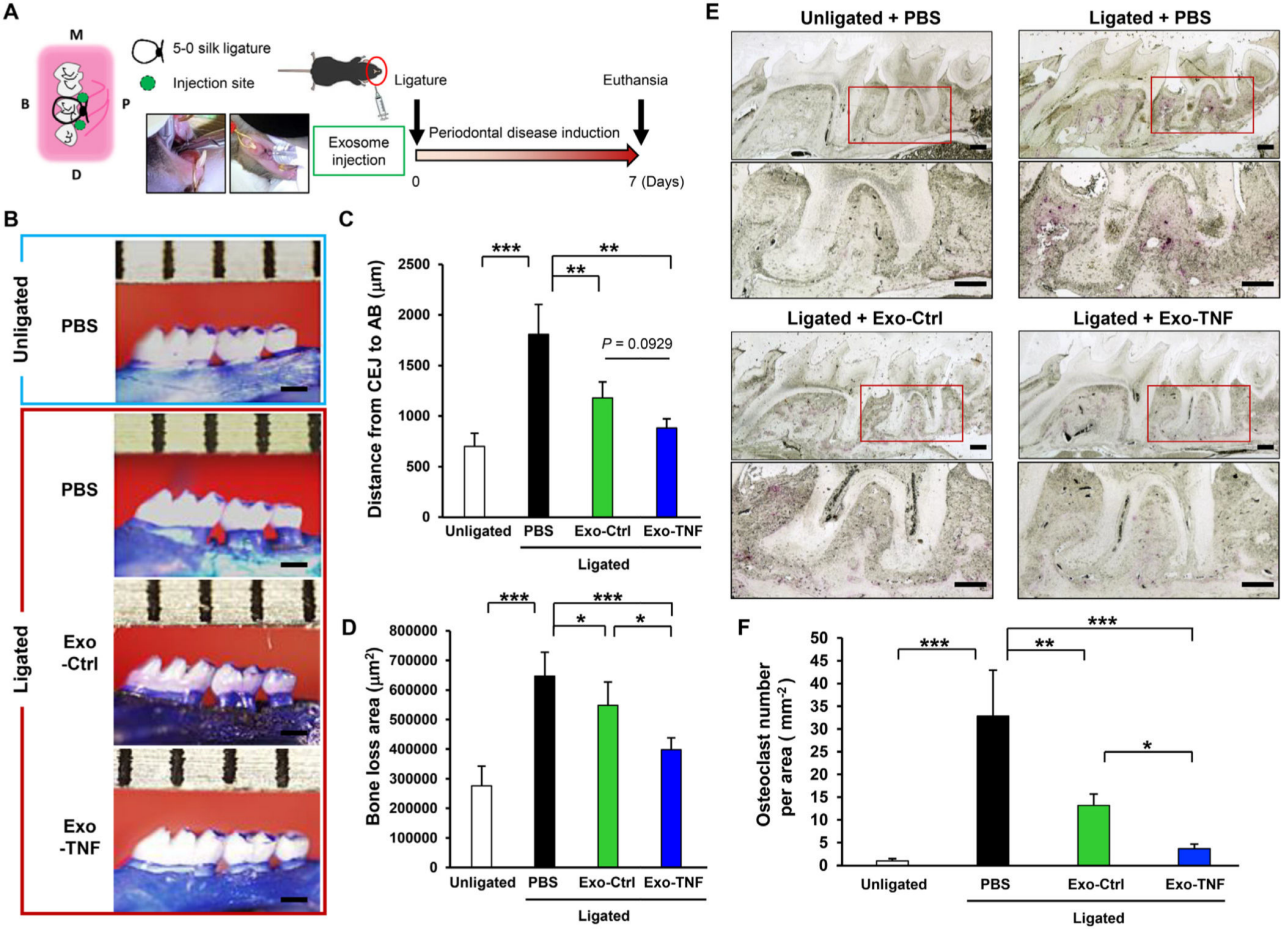
Therapeutic effect of GMSC-derived exosomes on periodontal bone loss in mice **(A)** Schematic illustration for ligature-induced periodontitis model and local administration of exosomes. A 5–0 silk ligature was tied around the maxillary second molar in C57BL/6 mice on day 0, and either placebo (PBS), GMSC-derived exosomes (Exo-Ctrl), or TNF-*α*-preconditioned GMSC-derived exosomes (Exo-TNF) (20 *μ*g) suspended in PBS (20 *μ*L) were simultaneously injected as illustrated. **(B)** Representative images of the maxillae in each treatment group at day 7 after ligature placement. **(C, D)** Periodontal bone resorption analysis. **(C)** The distance from the cementoenamel junction (CEJ) to the pinnacle of the alveolar bone (AB) was defined as the sum of distances from five predetermined points to assess periodontal bone loss using an Olympus DP72 digital camera. **(D)** The area of palatal alveolar bone loss around the 3 molars was pictured and measured using the Olympus DP-11BSW software. **(E)** TRAP staining for the presence of osteoclasts from periodontal tissue. Bone resorption was confirmed by the detection of osteoclasts. **(F)** The number of TRAP-positive osteoclasts per area (mm^2^) was counted. TRAP-positive multinucleated cells (osteoclasts) were enumerated from two random coronal sections of the ligated molar from five female mice in each group and the average number with SD from the total 10 sections/group was obtained. *p < 0.05, **p < 0.01, ***p < 0.001. Error bars represent means ± SD. (*n* = 10 per group) Statistical analyses were performed using one-way analysis of variance (ANOVA) with Bonferroni correction. Scale bar = 500 *μ*m.

Preconditioning of GMSCs with TNF-*α* enhanced macrophage polarization toward an anti-inflammatory (M2) phenotype, suggesting that this negative feedback loop could be potentially useful for the regulation of inflammatory bone loss. To test this hypothesis, we employed ligature-induced periodontitis model and the protective effects of GMSC-derived exosomes from inflammatory bone loss were examined using a split-mouth experimental design (Fig. 6A). At day 7 after ligation, severe alveolar bone loss was observed around the ligated 2nd molar, compared to unligated groups. In contrast, local injection of exosomes into the gingiva significantly reduced the bone resorption induced by the ligation, meanwhile the opposite control sites showed similar bone heights [distance from the cementoenamel junction (CEJ) to the alveolar bone crest (ABC)] at baseline (Fig. 6B). Distance from CEJ to ABC in the Exo-Ctrl group decreased to approximately half of the ligated control (PBS). Compared with Exo-Ctrl, Exo-TNF treatment found to be more effective with a clear statistical trend (*P* = 0.09 < 0.1) (Fig. 6C). When the volume of total bone loss was calculated, significant difference was observed between Exo-Ctrl and Exo-TNF groups (Fig. 6D). The number of TRAP-positive osteoclasts in the alveolar bone was significantly lower in mice treated with Exo-TNF than in mice treated with Exo-Ctrl, while it was significantly higher in ligated control (PBS) mice (Fig. 6E and F).

### 3.6. GMSC-secreted exosomes regulate the RANKL/OPG system by targeting Wnt5a-mediated RANKL expression

**Fig. 7.**
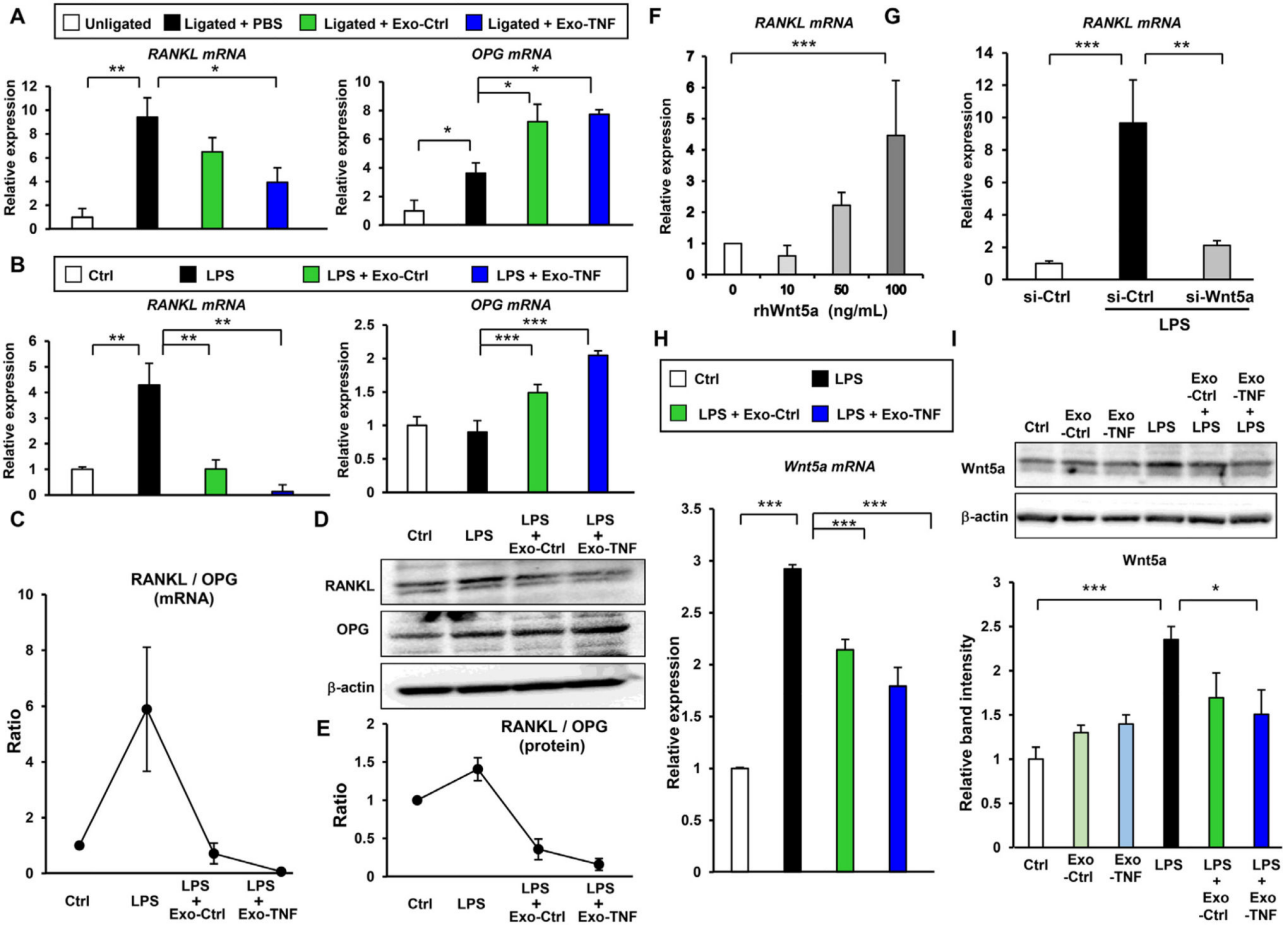
GMSC-derived exosomes regulate the RANKL/OPG ratio by targeting Wnt5a-mediated RANKL expression **(A)** Effect of GMSC-derived exosome injection on RANKL and OPG mRNA expression in ligature-induced mouse periodontitis model. Gingival tissues around the second maxillary molar on day 7 **Fig. 5)** were collected and total RNA were extracted for RANKL (**left**) and OPG (**right**) mRNA detection. Relative mRNA expressions were normalized to *GADPH*. **(B)** Effects of GMSC-derived exosomes on LPS-induced RANKL (**left**) and OPG (**right**) mRNA expression in human periodontal ligament **(**PDL) cells. Cells were incubated with 5 *μ*g/mL of GMSC-derived exosomes (Exo-Ctrl) or TNF-*α*-preconditioned GMSC-derived exosomes (Exo-TNF) for 12 h, followed by stimulation with LPS (5 *μ*g/mL) for 6 h. Relative mRNA expression was normalized to that of *GADPH*. **(C)** Effect of GMSC-derived exosomes on RANKL/OPG mRNA ratio in PDL cells was evaluated by calculating mRNA expression levels in **(B). (D)** Western blot analysis showing the expression of RANKL and OPG. PDL cells were incubated with 5 *μ*g/mL of Exo-Ctrl or Exo-TNF for 12 h, followed by stimulation with LPS (5 *μ*g/mL) for 48 h. **(E)** Effect of GMSC-derived exosomes on RANKL/OPG ratio based on protein expression in PDL cells as in (D). Results were obtained from three independent experiments. **(F)** Effect of Wnt5a on RANKL mRNA expression in human PDL cells. Cells were stimulated with the indicated amount of recombinant Wnt5a for 24 h and qRT-PCR was performed to assess RANKL mRNA expression. **(G)** Effect of Wnt5a knockdown on LPS-induced RANKL mRNA expression in PDL cells. PDL cells were transfected with siRNA for 24 h, followed by stimulation with LPS (5 *μ*g/mL) for 6 h, and qRT-PCR was performed. Control siRNA (si-Ctrl), Wnt5a siRNA (si-Wnt5a). **(H, I)** Effect of GMSC-derived exosomes on LPS-induced Wnt5a expression was assessed using qRT-PCR **(H)** and western blotting **(I). (H)** PDL cells were treated with 5 *μ*g/mL of LPS in the presence or absence of 5 *μ*g/mL of GMSC-derived exosomes (Exo-Ctrl) or TNF-*α*-preconditioned GMSC-derived exosomes for 1 h. **(I)** PDL cells were treated with 5 *μ*g/mL of Exo-Ctrl, Exo-TNF, LPS alone, and LPS in the presence of Exo-Ctrl or Exo-TNF for 3 h (**upper**). Relative Wnt5a protein expressions were measured by quantifying the density of bands and normalized against *β*-actin using the MultiGauge software (**lower**). *p < 0.05, **p < 0.01, ***p < 0.001. Error bars represent means ± SD. Statistical analyses were performed using one-way analysis of variance (ANOVA) with Bonferroni correction.

RANKL/OPG system is an essential regulator of osteoclastogenesis and bone resorption. Therefore, we examined the expression of RANKL and OPG in the gingival tissue from an experimental periodontitis mice model. qRT-PCR analysis revealed that treatment of both Exo-Ctrl and Exo-TNF resulted in reduced expression of RANKL mRNA, while the expression of OPG mRNA was significantly up-regulated (Fig. 7A). Since periodontal ligament (PDL)–alveolar bone interface plays a critical role in periodontal bone homeostasis [40], we examined the effect of GMSC-derived exosomes on RANKL/OPG signaling in PDL cells. LPS-induced RANKL expression was significantly inhibited in the presence of GMSC-derived exosomes preconditioned with or without TNF-*α*, while OPG expression was up-regulated as well (Fig. 7B). Consequently, GMSC-derived exosomes restored the increased RANKL/OPG ratio in the LPS-stimulated PDL cells, and it was lowered by Exo-TNF treatment as well (Fig. 7C). This was reflected in RANKL protein expression (Fig. 7D and E). To explore the underlying mechanism for GMSC-derived exosome-mediated RANKL inhibition in PDL cells, we investigated the involvement of Wnt signaling, which plays an essential role in osteoclastogenesis [41]. Wnt5a stimulation up-regulated RANKL mRNA expression in a dose-dependent manner (Fig. 7F), while Wnt5a knockdown abrogated LPS-induced RANKL expression in PDL cells (Fig. 7G). To assess the effect of GMSC-derived exosomes on Wnt5a expression, PDL cells were stimulated with LPS in the presence or absence of GMSC-derived exosomes. qRT-PCR revealed that LPS stimulation significantly up-regulated Wnt5a mRNA expression, while it was diminished by the Exo-Ctrl and Exo-TNF treatment (Fig. 7H). Western blot analysis further demonstrated that Exo-Ctrl and Exo-TNF treatments significantly down-regulated LPS-induced Wnt5a expression, although exosome stimulation alone did not affect Wnt5a expression in the absence of LPS (Fig. 7I). These results indicated that LPS-induced RANKL expression was regulated by Wnt5a, and GMSC-derived exosomes potentially targeted Wnt5a to induce RANKL suppression in PDL cells.

### 3.7. miR-1260b is essential for exosome-mediated RANKL inhibition

**Fig. 8.**
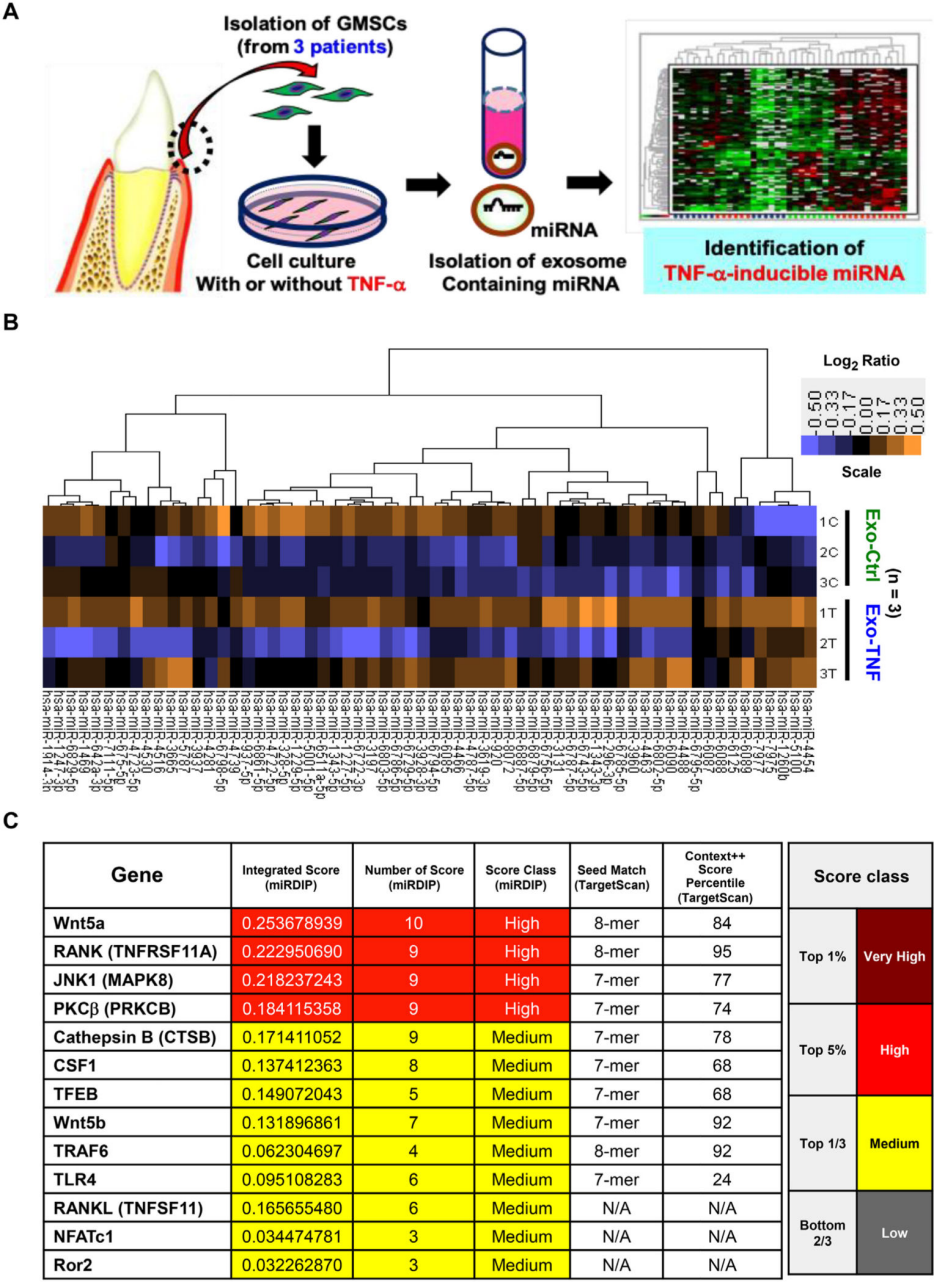
Identification of TNF-*α* inducing exosomal miRNAs in GMSCs **(A)** Experimental strategy for the identification of TNF-*α* inducing exosomal miRNA derived from GMSCs. **(B)** Heat map and unsupervised hierarchical clustering. Clustering was performed on GMSC-derived exosomes from three patient-derived samples. Exo-Ctrl (“C”) and Exo-TNF (“T”) for three patients (1, 2, and 3) were abbreviated as “1–3C” and “1–3T”. Each row represents one miRNA, and each column represents one sample. The miRNA clustering tree is shown on the upper side. The color scale illustrates the relative expression levels of miRNA across all samples: yellow color represents an expression level above mean and purple color represents expression lower than the mean. **(C)** Potential of hsa-miR-1260b in regulating osteoclastogenesis-related genes as predicted from the mirDIP and TargetScan Databases. (For interpretation of the references to color in this figure legend, the reader is referred to the web version of this article.)

**Fig. 9.**
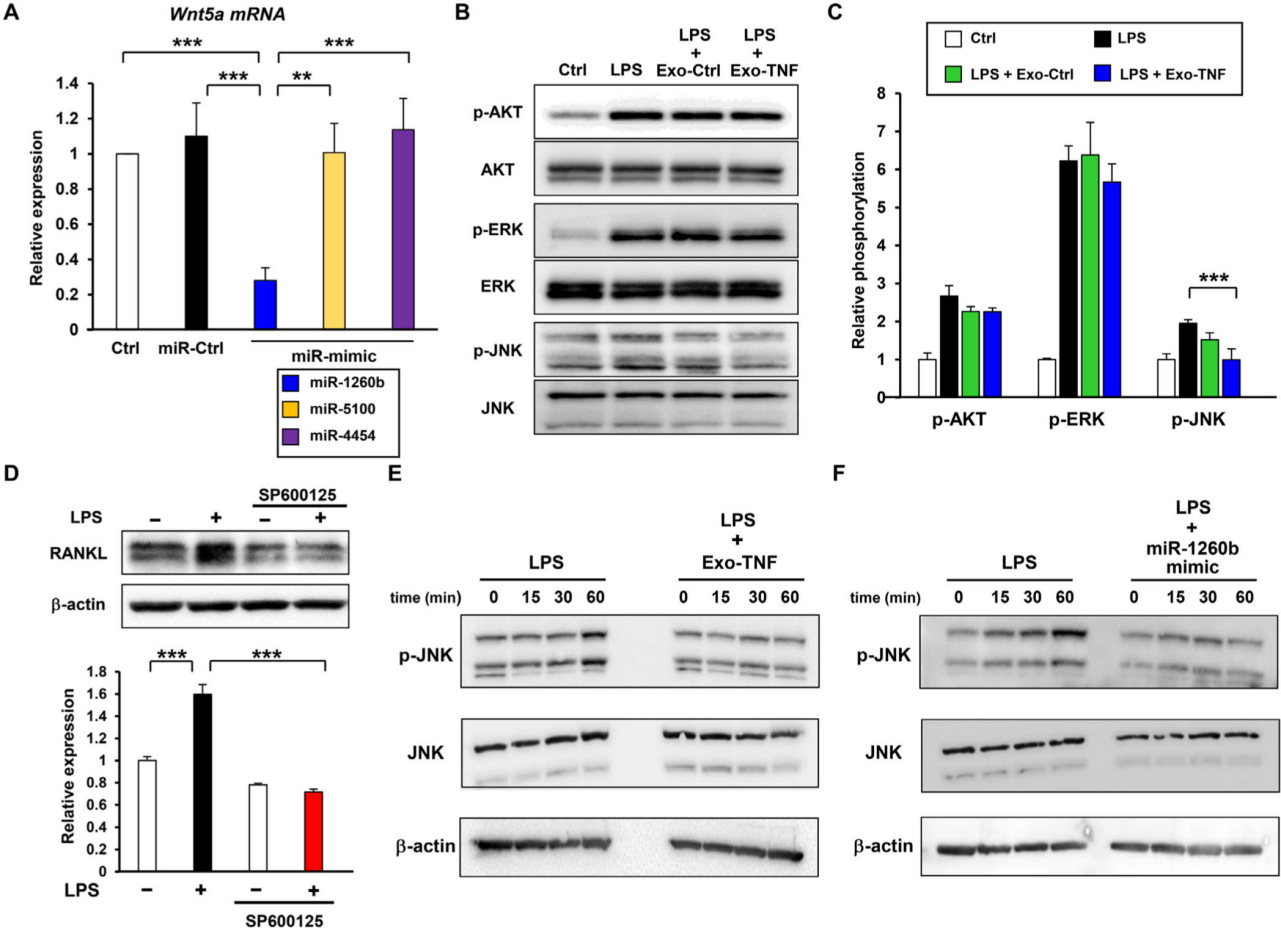
Identification of RANKL targeting exosomal miRNA induced by TNF-*α* treatment in GMSC **(A)** Validation of novel TNF-*α* inducible exosomal miRNA-mediated Wnt5a gene silencing was assessed using qRT-PCR. PDL cells were transfected with 20 nM of miRNA mimics for potent TNF-*α*-inducible exosomal miRNAs as indicated. **(B) and (C)** Effect of GMSC-derived exosomes on LPS-induced signaling. PDL cells were treated with 5 *μ*g/mL of LPS in the presence or absence of 5 *μ*g/mL of GMSC-derived exosomes (Exo-Ctrl) or TNF-*α*-preconditioned GMSC-derived exosomes for 15 min to detect AKT, 45 min to detect ERK, and 60 min to detect JNK phosphorylation, respectively, using western blot analysis **(B). (C)** Relative phosphorylation was measured by quantifying the density of each corresponding Pan/Phospho-bands using the MultiGauge software. **(D)** Effect of JNK inhibition on LPS-induced RANKL expression in PDL cells. PDL cells were stimulated with LPS (5 *μ*g/mL) for 48 h with or without pre-treatment with SP600125 (10 *μ*M) for 1 h. Relative RANKL protein expressions were measured by quantifying the density of bands and normalized against *β*-actin using the MultiGauge software. **(E, F)** Inhibition of LPS-mediated JNK signaling using Exo-TNF and miR-1260b. PDL cell were stimulated with LPS for 60 min in the presence or absence of Exo-TNF **(E),** or after transfection using miR-1260b mimics **(F)**. Time-course phosphorylation of JNK was monitored using western blotting. **p < 0.01, ***p < 0.001. Error bars represent means ± SD. Statistical analyses were performed using one-way analysis of variance (ANOVA) with Bonferroni correction.

To gain further insight into GMSC-derived exosome-mediated RANKL inhibition, we postulated that exosomal miRNAs could be transferred into PDL cells to interfere with the Wnt5a mRNA expression. Moreover, Exo-TNF inhibited periodontal bone loss in mice model more efficiently than Exo-Ctrl. This led us to hypothesize that TNF-*α* stimulation might change the composition of GMSC-derived exosome cargo, and some specific miRNAs in Exo-TNF might account for the enhanced inhibitory effect on osteoclastogenesis. Thus, we performed miRNA microarray screening by comparing the effect of TNF-*α* preconditioning among three patient-derived GMSCs (Fig. 8A). Following data normalization, we found 655 universal differentially expressed miRNAs in Exo-TNF compared to Exo-Ctrl. To exclude the individual differences, we extracted substantially up-regulated and downregulated 62 miRNAs based on the average expression of all samples. A heat map for unsupervised hierarchical clustering performed on these differentially expressed 62 miRNAs is shown in Fig. 8B. We further screened for significantly up-regulated miRNAs using the microRNA Data Integration Portal (mirDIP) and TargetScan database to identify miRNAs that could be potentially associated with osteoclastogenesis ( Fig. 8C). Specifically, miR-1260b was ranked in the top three of most highly up-regulated miRNAs by using TNF-*α* preconditioning, and the TargetScan database analysis revealed that hsa-miR-1260b could be associated with Wnt signaling genes, including WNT2B, WNT5A, WNT5B, dickkopf WNT signaling pathway inhibitor 2 (DKK2), WNT9B, and WNT8. To validate these results, PDL cells were transfected with miRNA mimics for miR-1260b and two other top up-regulated miRNAs (miR-5100 and miR-4454) following TNF-*α* preconditioning. qRT-PCR demonstrated that miR-1260b specifically decreased the expression of Wnt5a mRNA (Fig. 9A), thereby indicating that Exo-TNF containing miR-1260b interfered with Wnt5a mRNA expression, thus inhibiting RANKL expression.

We further examined the effect of GMSC-derived exosomes on LPS-induced signaling pathways in PDL cells, which is essential for RANKL expression. After confirming that LPS-mediated phosphorylation of AKT, ERK, and JNK peaked at 15 min, 45 min, and 60 min, phosphorylation levels of these signaling in the presence of Exo-Ctrl and Exo-TNF at each time point were examined. Exo-TNF specifically decreased the phosphorylation of JNK, whereas AKT and ERK pathways were not affected by GMSC-derived exosomes (Fig. 9B and C). Moreover, we found that LPS-induced RANKL expression was abrogated by specific inhibitor for JNK, SP600125 (Fig. 9D), thereby indicating that Exo-TNF suppressed RANKL expression via JNK signaling. To validate if this effect was mediated by Exo-TNF-containing miR-1260b, time course inhibition of Exo-TNF and miR-1260b on LPS-mediated JNK activation was monitored for 60 min. Both pretreatment of Exo-TNF and transfection of miR-1260b significantly suppressed LPS-induced phosphorylation of JNK in PDL cells (Fig. 9E and F). These results suggested that Exo-TNF containing miR-1260b was critical for RANKL inhibition by targeting Wnt5a expression as well as JNK signaling.

## 4. Discussion

MSC-based cell therapy has entered the clinical trial phase for the purpose of tissue regeneration and the treatment of autoimmune disorder. According to the National Institutes of Health’s clinical trial registry, more than 700 clinical trials for MSCs therapy are currently in progress throughout the world (http://clinicaltrials.gov). Although animal models and clinical case reports have demonstrated that MSCs can be successfully applied to periodontal defects [42], only two clinical trials have been registered in ClinicalTrials.gov for periodontal disease. The reason for this may not only be attributed to the restrictions on ethical regulation, but may also be attributed to the time and cost for the preparation of large quantities of cells. Considering that therapeutic effect of MSCs largely depends on the paracrine efficiency of MSCs [4] and the advantages of the use of GMSCs including easier isolation from small piece of gingival tissue (~2 × 2 mm^2^ ), rapid cell proliferation, and the ability to secrete large amount of exosomes, we speculated that GMSC-derived exosome-based therapy could be ideal for clinical use.

While CD73 is widely considered as a classical marker of MSCs [43], its essential role is to mediate immune suppression by enzymatically converting ATP to adenosine. Exosomal expression of CD73 was first identified in human cancer cells, which contributed to extracellular adenosine production, thereby leading to tumor growth and metastasis [44]. More recently, expression of CD73 on MSC-secreted exosomes has also been reported [45]. In the present study, we not only showed that GMSC-derived exosomes express CD73, but also identified for the first time that TNF-*α* preconditioning of GMSCs enhanced CD73 expression on these exosomes. Interestingly, treatment with TNF-*α* in GMSCs significantly increased CD73 mRNA expression, while it did not change in endothelial cells [46] and astrocytes [47], suggesting that induction of CD73 by TNF-*α* stimuli was GMSC-specific. We also found that TNF-*α* (100 ng/mL) stimulation induced larger amount of exosome secretion (2.7-fold) in GMSCs. It was reported that TNF-*α* was able to slightly increase the GMSCs proliferation at a low concentration (5–10 ng/mL), while a high concentration (100 ng/mL) of TNF-*α* slightly decreased its proliferation after 48 h [48], of which the latter corresponded to our preconditioning protocol. Exosome production is regulated by Rab27, a family of small GTPases [49], and the expression of Rab27A is regulated by NF-*κ*B signaling [50]. Therefore, we speculate that TNF-*α* induces Rab27 activation via the NF-*κ*B pathway to promote exosome release, thereby yielding prominent effect that outweighs decreased cell viability.

Emerging evidence has shown the immunoregulatory effects of MSC-derived exosomes [51], but few reports have addressed MSC-macrophage cross talk, by which anti-inflammatory M2 macrophage polarization is induced [52, 53]. In this study, we demonstrated that GMSC-derived exosomes significantly promoted recipient macrophages toward M2 polarization, and the effect was further enhanced by TNF-*α* preconditioning of GMSCs. Previous studies have demonstrated that injury-based preconditioning (TNF-*α*, LPS, and hypoxia) of MSCs can increase the production of growth factors [54], and LPS-preconditioned MSC-derived exosomes can induce M2 polarization in THP-1 cells [55]. Similarly, we reported that MSCs stimulated with TNF-*α* enhanced production of IL-1RA-containing exosomes [30], that IFN-*γ* improved dentin regeneration activity [31], and that ASA promoted osteogenesis [56]. Considering these results, we compared the effect of 100 ng/mL of TNF-*α*, IFN-*γ*, LPS, and aspirin on GMSC-derived exosomes and found that only TNF-*α* treatment significantly polarized macrophage phenotype from M1 to M2. In the current study, not only M1 macrophage generated from PBMC, but also GMSC-derived exosomes stimulated macrophage included CD86^+^ CD206^+^ cell population (Fig. 5D). In such double stained macrophages, expression of CD206^+^ was partially accounted by M-CSF-induced macrophages differentiation procedure, since M-CSF is a potent M2 macrophage inducer [57]. Conversely, CD86^+^ cells were considered to be remnant from M1 macrophage induction. However, GMSC-derived exosomes significantly increased CD86^−^CD206^+^ M2 macrophage polarization from CD86^+^ CD206^−^ M1 macrophages, and we further demonstrated that expression level of exosomal CD73 was associated with the ability of exosomes to switch M1 macrophage into M2 phenotype.

Recent studies have demonstrated that infused GMSCs exerted prominent immunoregulation via CD39/CD73/adenosine signaling [58], and endogenous CD73 activity of GMSCs contributed to macrophage differentiation [59]. However, it is still unclear whether CD73 not anchored to the cell membrane is a good adenosine manufacturer. Although neutralizing anti-CD73 Ab-treated exosomes decreased the induction of M2 marker expression suggesting that CD73 could be anchored on the surface of exosome, the precise molecular mechanism mediated by exogenously added CD73 should be validated in future study. While the therapeutic effect of MSC-exosomes on cutaneous regeneration is widely accepted [60], induction of M2 macrophage plays a critical role in the resolution of inflammation and tissue remodeling in wound healing model [61]. Consistently, our current study also demonstrated that injection of Exo-TNF significantly accelerated wound healing and closure together with accelerated M2 macrophage infiltration. Taken together, these findings support the notion that manipulating the differentiation of plastic macrophage toward M2 phenotype may provide clinical strategies for not only better wound healing, but also for the treatment of inflammatory disorders.

Given the close relationship between inflammation and bone resorption in periodontal disease [62], our findings show that significant inhibition of bone loss by GMSC-derived exosomes in a mouse periodontal model can be explained by the anti-inflammatory effects of exosomes, including TNF-*α*-mediated exosomal CD73 up-regulation and subsequent M2 macrophage infiltration *in vivo*. Recent studies have also reported that transplantation of periodontal ligament stem cells (PDLSCs) which possess immunomodulatory properties [63] similar to GMSCs, could induce macrophage polarization toward M2 to enhance periodontal regeneration [64]. Furthermore, PDLSCs co-cultured with M2 macrophages enhanced cementoblast differentiation [65], suggesting the potential of M2-macrophage-mediated periodontal regeneration. Exosome-mediated cell differentiation and reprogramming can be accomplished through mutual communication between MSCs and tissue-injured cells [66]. Except for the direct effect of M2 macrophage on PDLSCs, whether Exo-TNF can contribute to periodontal tissue regeneration needs to be further investigated. Overall, we demonstrated that injection of Exo-TNF significantly decreased TRAP-positive osteoclasts in the mouse periodontitis model. The possible roles of CD73 and adenosine in bone homeostasis have been reported [67]. The expression of CD73 is regulated by the Wnt-*β*-catenin pathway, which is a critical signaling pathway in bone metabolism [68] and CD73 KO mice exhibited osteopenia [69], thus indicating that the activity of CD73 is essential for osteogenesis. Most recent studies have demonstrated that adenosine regulates not only osteogenesis, but also osteoclastogenesis via A_2B_ R, and osteoporosis model mice exhibits decreased adenosine levels [70]. Therefore, we speculated that CD73 was one of the key molecules in Exo-TNF that suppressed periodontal bone loss. In support of this notion, Luo et al. demonstrated that osteoclast precursors co-cultured with GMSC partially suppressed osteoclast differentiation via CD39-CD73-adenosine pathway [71].

The RANKL/OPG ratio is a well-recognized biomarker for periodontal tissue destruction [72]. During periodontitis, both PDL cells and osteoblasts increase the RANKL/OPG ratio to participate in osteoclastic bone resorption [12, 73]. PDL tissue surrounds the root of the tooth and anchors it to the tooth socket surrounded by the bone, and plays a pivotal role in tooth maintenance [74]. Thus, PDL cells are involved in the regulation of osteoclastogenesis in the alveolar bone through the release of RANKL [40]. We showed that Exo-TNF treatment decreased the RANKL/OPG ratio both *in vitro* and *in vivo*. Another interesting result was that enhanced expression of OPG mRNA was observed in mouse ligated gingival tissue, while LPS-stimulation had no effect in PDL cells. To confirm OPG producing cells in gingival tissue, we isolated gingival fibroblasts from mice (mGFs) and OPG and RANKL mRNA expression in LPS-stimulated mGFs were analyzed. LPS-stimulation significantly increased the expression of OPG mRNA, while RANKL mRNA was unchanged in mGFs (Fig. S6). These results suggest that mGFs were central producer of OPG in ligature-induced mouse periodontal model. In support of this notion, previous report demonstrated that human gingival fibroblasts (HGFs) produce OPG and it was enhanced by LPS-stimulation to protect from bacterial challenge [75].

We further investigated precise molecular mechanism of Exo-TNF–mediated regulation of RANKL. While toll-like receptor plays a critical role in microbial infection-induced periodontal inflammation [76], emerging evidence indicates that Wnt signaling pathways also play essential roles in host response to inflammation [77]. The Wnt/TLR-axis is involved in certain inflammatory disorders [78], such as sepsis, Alzheimer’s disease [79], obesity, and type 2 diabetes [80]. Specifically, recent studies have shown that the enhanced Wnt5a expression in the gingival tissue is associated with periodontal inflammatory process [81, 82]. As an inflammatory mediator, the expression of Wnt5a is regulated by TLR-mediated NF-kB signaling [83] and involved in osteoclastogenesis [84]. Collectively, our results suggest that LPS stimulation targets TLR-mediated signaling and induces Wnt5a expression, and this induction is downregulated by Exo-TNF treatment, thereby inhibiting RANKL expression in PDL cells.

It is widely accepted that enriched miRNAs in MSC-exosomes can post transcriptionally regulate gene expression [85], thereby leading to phenotypic modification and reprogramming of recipient cells [66]. Moreover, the secretion of exosomes is enhanced in cells exposed to a stress environment including inflammatory cytokines, hypoxia, and the expression profiles of exosomal miRNAs change distinctively according to these stimuli [86]. However, whether exosomal miRNA profile is modulated by TNF-*α* in GMSCs remains unclear. To further explore the possibility that the TNF-*α*-regulated exosomal miRNA might prevent periodontal bone loss, we performed miRNA array analysis. In this study, we demonstrated that miR-1260b, which was significantly up-regulated by TNF-*α*-stimulation, specifically targeted Wnt5a and JNK to downregulate RANKL expression. Wnt5a has been reported to enhance RANKL expression via phosphorylation of JNK [87], which is an essential signaling mechanism for the activation of osteoclastogenesis [88]. Similarly, we demonstrated that not only Exo-TNF treatment, but also transfection of miR-1260b mimicked in PDL cells significantly reduced phosphorylation of JNK. The impact of MSC-derived exosomes on signaling pathways have also been reported namely, bone marrow somatic cell-derived exosomes inhibited the JNK pathway [89], while MSC-derived exosomal CD73 contributed to the activation of AKT and ERK [90]. Considering the possibility that LPS-stimulation alone induces saturation of phosphorylation of AKT and ERK, further activation of these molecules by external exosomes have not been observed in our study. Database analysis revealed that miR-1260b could regulate osteoclastogenesis-associated genes, and Wnt5a and JNK1 were predicted to be the major targets (Fig. 8C). It should be noted that other miR-1260b targeting candidate genes included RANK, TNF receptor-associated factor 6 (TRAF6), and NFATc1, all of which were the master inducer genes for osteoclast differentiation [91]. Protein kinase C-*β* (PKC *β*) and transcription factor EB (TFEB) are involved in RANKL-mediated bone resorption in osteoclasts [92], and CSF-1 plays a key role in osteoclast recruitment [93]. Thus, our finding that the injection of Exo-TNF significantly decreased TRAP-positive cells in our mouse periodontitis model strongly suggests that miR-1260b is involved in the regulation of osteoclastogenesis. Furthermore, considering that miR-1260b has been reported to be downregulated in the gingival tissue from periodontitis [94], it may suggest another mechanism by which miR-1260b inhibits alveolar bone resorption.

Previous studies have shown that miR-146a, miR-125a, and miR-145–5p modulates macrophages from a pro-inflammatory M1 to an anti-inflammatory M2 phenotype [95]. Meanwhile, let-7 miRNA family targets NF-kB signaling [96] and exosomes from LPS-preconditioned MSCs enhance let-7b to induce M2 polarization in THP-1 cells [55]. In our current study, exosomes from LPS-stimulated GMSCs did not enhance M2 macrophage polarization, and the expression of these miRNAs was almost unchanged as observed in microarray data. This discrepancy may be due to differences in the origin of MSCs, and further investigation is required to identify and characterize other downstream target molecules of exosomal miRNAs in TNF-*α*-stimulated GMSCs. It should be noted that miR-1260b was one of the few miRNAs which was universally up-regulated among three patient-derived specimens. Therefore, miR-1260b may be a target for miRNA mimic-based treatment against inflammation-induced bone loss.

## 5. Conclusions

Our present study reveals that therapeutic effect of TNF-*α*-preconditioned GMSC-derived exosomes was induced by the up-regulation of CD73 and miR-1260b. TNF-*α* promoted CD73 expression on exosomes to induce M2 macrophage polarization, thus contributing to the resolution of inflammation and preventing bone loss in the periodontal tissue. Exosomal miR-1260b was essential for the inhibition of osteoclastogenesis via the Wnt5a-mediated RANKL pathway. These latest findings and the proposed therapeutic strategy are summarized (Schema 1). Taken together with its unique advantages including easy accessibility and prominent exosome productive ability, GMSC is one of the most ideal exosome sources for clinical applications. Collectively, TNF-*α*-preconditioned GMSC-derived exosomes may be a promising therapeutic tool against periodontitis and other inflammatory disorder leading to bone loss.

## Supplementary Material

1

## Figures and Tables

**Schema 1.**
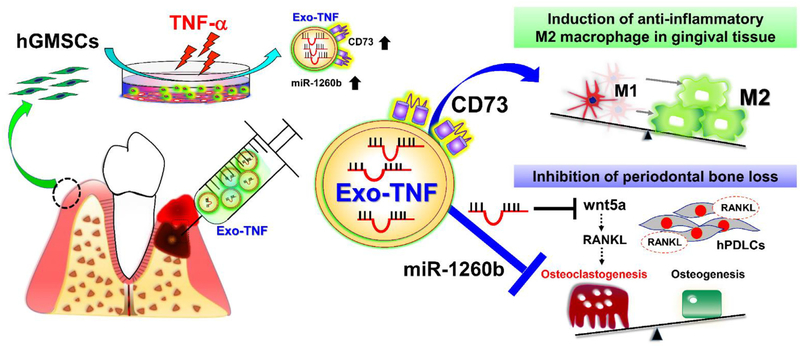
Proposed therapeutic strategy for periodontal disease using exosomes derived from TNF-*α*-treated GMSCs.

## Data Availability

The data used to support the findings of this study are available from the corresponding author on request.

## Statement of significance

Therapeutic effect of mesenchymal stem cell (MSC) largely depends on the paracrine efficiency of MSC, and gingiva-derived MSC (GMSC) have unique capacity to secret large amount of exosomes and, moreover, are easier to isolate. Recent studies indicated that appropriate preconditioning of MSC with disease-related stimuli can optimize the exosomal protein or miRNA contents which may efficiently support the healing and repair of specific diseases. In this study, we examined the therapeutic effects of TNF-*α* preconditioned-GMSC-derived exosomes on periodontal disease, and show for the first time an optical protocol for MSC-preconditioning targeting both exosomal protein- and miRNA-mediated attenuation of inflammation and osteoclastogenesis. These findings prove a therapeutic strategy for patients not only with periodontitis but with other inflammatory osteoimmune disorders.
