# Corrigendum to “Exosomes from TNF-α-treated human gingiva-derived MSCs enhance M2 macrophage polarization and inhibit periodontal bone loss” [Acta Biomaterialia 2021, 122, 306-324]

**DOI:** 10.1016/j.actbio.2024.11.029

**Published:** 2024-12-01

**Authors:** Yuki Nakao, Takao Fukuda, Qunzhou Zhang, Terukazu Sanui, Takanori Shinjo, Xiaoxing Kou, Chider Chen, Dawei Liu, Yukari Watanabe, Chikako Hayashi, Hiroaki Yamato, Karen Yotsumoto, Urara Tanaka, Takaharu Taketomi, Takeshi Uchiumi, Anh D. Le, Songtao Shi, Fusanori Nishimura

**Affiliations:** a Department of Periodontology, Division of Oral Rehabilitation, Faculty of Dental Science, Kyushu University, Fukuoka, Japan; b Department of Anatomy and Cell Biology, University of Pennsylvania School of Dental Medicine, Philadelphia, PA, USA; c Department of Oral and Maxillofacial Surgery and Pharmacology, University of Pennsylvania School of Dental Medicine, PA, USA; d South China Center of Craniofacial Stem Cell Research, Guanghua School of Stomatology, Sun Yat-sen University, Guangdong, China; e Department of Orthodontics, Peking University School and Stomatology, Peking, China; f Dental and Oral Medical Center, Kurume University School of Medicine, Fukuoka, Japan; g Department of Clinical Chemistry and Laboratory Medicine, Graduate School of Medical Sciences, Kyushu University, Fukuoka, Japan

The authors regret to report that a duplicate image was published in Fig. 3D. The image of Exo-TNF Day 0 was the same as PBS Day 5. Below is a corrected full Fig. 3 with the original figure caption. This error does not affect the results of Fig. 3 or the conclusions of the study. The authors sincerely apologize for any inconvenience caused.

## Figures and Tables

**Fig. 3.**
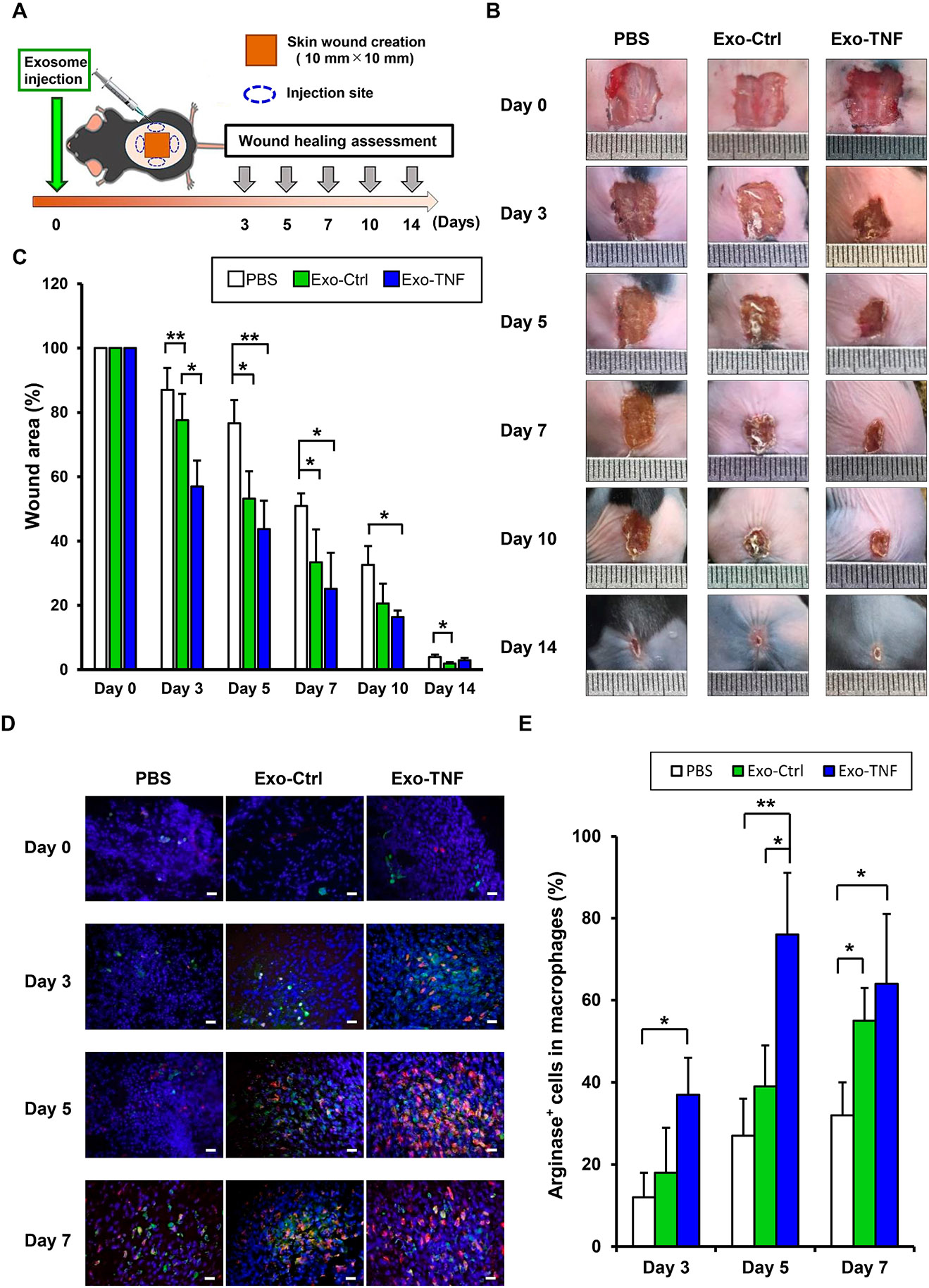
Therapeutic effect of GMSC-derived exosomes on skin wound healing in mice (A) Schematic illustration for skin wound healing mouse model and local administration of exosomes. (B) Representative healing process of cutaneous wounds in each group. A full-thickness 10 mm2 wound was made in C57BL/6 mice. Either placebo (PBS) or GMSC-derived exosomes (Exo-Ctrl), or TNF-α-preconditioned GMSC-derived exosomes (Exo-TNF) (200 μg) dissolved in PBS (200 μL) were injected subcutaneously as illustrated. (C) Wound closure kinetics (n = 5). The percentage of wound area was calculated as: (area of original wound – area of measured wound)/area of original wound × 100. (D) M2 macrophage detection in each wound. Frozen sections of full-thickness incisional skin wounds from mice after treatment with or without GMSC-derived exosomes for different days were immune-stained with DAPI (blue), F4/80 (green), and arginase-1 (red). Scale bar = 50 μm. (E) Quantification of the percentage arginase+ cells in F4/80+ cells according to immunofluorescence analysis. *p < 0.05, **p < 0.01. Error bars represent means ± SD. Data were analyzed using independent unpaired two-tailed Student’s t-tests.

