# Supplementary material for: Exosomes from TNF-α-treated human gingiva-derived MSCs enhance M2 macrophage polarization and inhibit periodontal bone loss
Source: Acta Biomater. Author manuscript; Available in PMC 2021 Mar 1. (PMC7897289; doi:10.1016/j.actbio.2020.12.046)
Supplement: 1 [file NIHMS1659012-supplement-1.docx]

**Supplementary material**

**Exosomes from TNF-α-treated human gingiva-derived MSCs enhance M2 macrophage polarization and inhibit periodontal bone loss**

Yuki Nakao^1#^, Takao Fukuda^1, 4#^, Qunzhou Zhang^2^, Terukazu Sanui^1^, Takanori Shinjo^1^, Xiaoxing Kou^3, 4^, Chider Chen^2, 3^, Dawei Liu^3, 5^, Yukari Watanabe^1^, Chikako Hayashi^1^, Hiroaki Yamato^1^, Karen Yotsumoto^1^, Urara Tanaka^1^, Takaharu Taketomi^6^, Takeshi Uchiumi^7^, Anh D. Le^2^, Songtao Shi^3, 4^ and Fusanori Nishimura^1^*

^1^ Department of Periodontology, Division of Oral Rehabilitation, Faculty of Dental Science, Kyushu University, Fukuoka, Japan

^2^ Department of Oral and Maxillofacial Surgery and Pharmacology, University of Pennsylvania School of Dental Medicine, PA, USA.

^3^ Department of Anatomy and Cell Biology, University of Pennsylvania School of Dental Medicine, Philadelphia, PA, USA

^4^ South China Center of Craniofacial Stem Cell Research, Guanghua School of Stomatology, Sun Yat-sen University, Guangdong, China

^5^ Department of Orthodontics, Peking University School and Stomatology, Peking, China

^6^ Dental and Oral Medical Center, Kurume University School of Medicine, Fukuoka, Japan

^7^ Department of Clinical Chemistry and Laboratory Medicine, Graduate School of Medical Sciences, Kyushu University, Fukuoka, Japan

**Table S1.**

Primer sequence used for quantitative RT-PCR

| **Gene** | **Forward primer** | **Reverse primer** |
| --- | --- | --- |
| ***human* LPL** | 5'-***CATTCCCGGAGTAGCAGAGT***-3' | 5'-***GGCCACAAGTTTTGGCACC***-3' |
| ***human* FABP-4** | 5'-**CTGGGCCAGGAATTTGACG**-3' | 5'-**CTCGTGGAAGTGACGCCTT**-3' |
| ***human Runx2*** | 5'-**GCGTCAACACCATCATTCTG**-3' | 5'-**CAGACCAGCAGCACTCCATC**-3' |
| ***human ALP*** | 5'-**GACAAGAAGCCCTTCACTGC**-3' | 5'-**AGACTGCGCCTGGTAGTTG**-3' |
| ***human* SOX9** | 5'-**AGACAGCCCCCTATCGACTT**-3' | 5'-**CGGCAGGTACTGGTCAAACT**-3' |
| ***human Runx2*** | 5'-**GCGTCAACACCATCATTCTG**-3' | 5'-**CAGACCAGCAGCACTCCATC**-3' |
| ***human COL2A1*** | 5'- **CCAGATGACCTTCCTACGCC**-3' | 5'-**TTCAGGGCAGTGTACGTGAAC**-3' |
| ***human OCN*** | 5'-**GGCGCTACCTGTATCAATGG**-3' | 5'-**TCAGCCAACTCGTCACAGTC**-3' |
| ***human COL2A1*** | 5'- **CCAGATGACCTTCCTACGCC**-3' | 5'-**TTCAGGGCAGTGTACGTGAAC**-3' |
| ***human* SOX9** | 5'-**AGACAGCCCCCTATCGACTT**-3' | 5'-**CGGCAGGTACTGGTCAAACT**-3' |
| ***human IL-10*** | 5'-**GTGATGCCCCAAGCTGAGA**-3' | 5'-**CACGGCCTTGCTCTTGTTTT**-3' |
| ***human TNF-α*** | 5'-**CCCAGGGACCTCTCTCTAATCA**-3' | 5'-**GCTTGAGGGTTTGCTACAACATG**-3' |
| ***human IL-1β*** | 5'-**ACGATGCACCTGTACGATCA**-3' | 5'-**TCTTTCAACACGCAGGACAG**-3' |
| ***human iNOS*** | 5'-**CAAGCCTACCCCTCCAGATG**-3' | 5'-**CATCTCCCGTCAGTTGGTAGGT**-3' |
| ***human CD39*** | 5'-**AGGTGCCTATGGCTGGATTAC**-3' | 5'-**CCAAAGCTCCAAAGGTTTCCT**-3' |
| ***human CD73*** | 5'-**GCCTGGGAGCTTACGATTTTG**-3' | 5'-**TAGTGCCCTGGTACTGGTCG**-3' |
| ***human RANKL*** | 5'-**AGAGCGCAGATGGATCCTAA**-3' | 5'-**TTCCTTTTGCACAGCTCCTT**-3' |
| ***human OPG*** | 5'-**ATGCAACACACGACAACATA**-3' | 5'-**GTTGCCGTTTTATCCTCTCT**-3' |
| ***human Wnt5a*** | 5'-**AGGGCTCCTACGAGAGTGCT**-3' | 5'-**GACACCCCATGGCACTTG**-3' |
| ***human GAPDH*** | 5'-**ATCAAGAAGGTGGTGAAGCAGG**-3' | 5'-**GTCATACCAGGAAATGAGC**-3' |
| ***mouse RANKL*** | 5'-**CGCTCTGTTCCTGTACTTTCG**-3' | 5'-**GAGTCCTGCAAATCTGCGTT**-3' |
| ***mouse OPG*** | 5'-**CCTTGCCCTGACCACTCTTAT-**3' | 5'-**CACACACTCGGTTGTGGGT**-3' |
| ***mouse GAPDH*** | 5'-**AATGTGTCCGTCGTGGATCTGA**-3' | 5'-**GTCATACCAGGAAATGAGC**-3' |

**
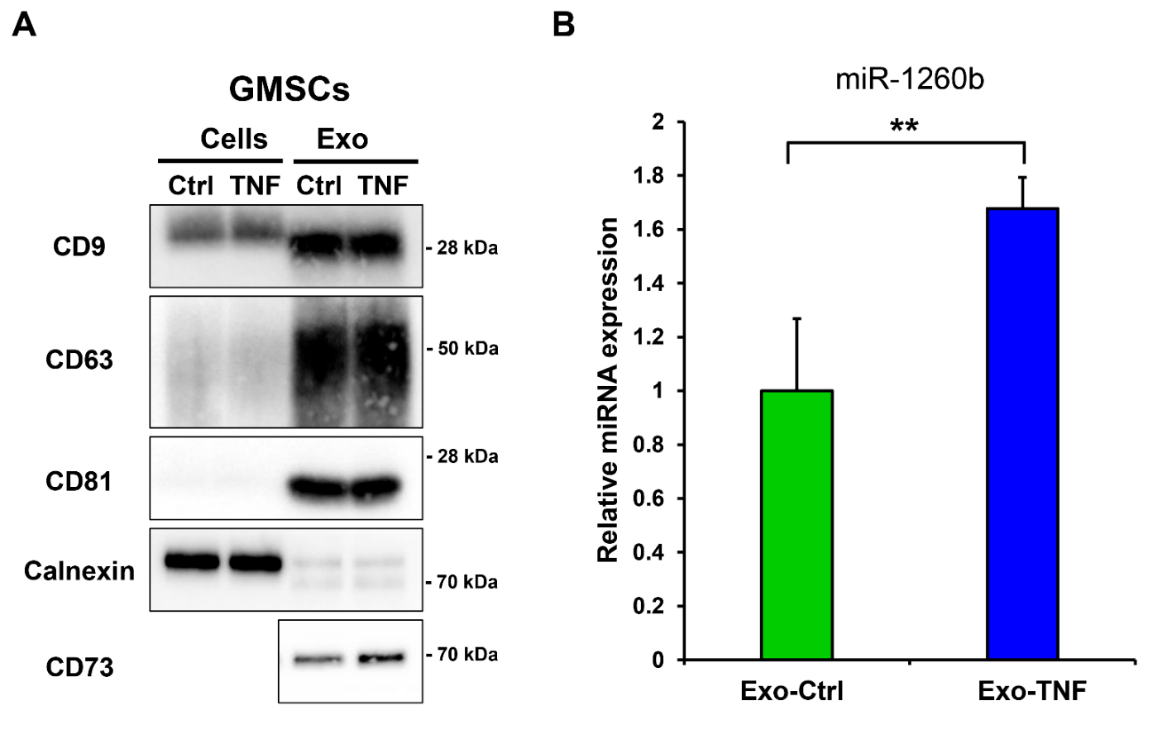
**

**Fig. S1. Characterization of GMSC-derived exosomes isolated by differential centrifugation.**

(A) Western blot analysis showing the expression of CD9, CD63, CD8, calnexin and CD73 in GMSCs (Cells), GMSC-derived exosomes (Exo-Ctrl) and TNF-α-preconditioned GMSC-derived exosomes (Exo-TNF). Protein samples from GMSC cell lysate with or without preconditioning and GMSC-derived exosomes were subjected to analyses. (B) The expression levels of miR-1260b in Exo-Ctrl and Exo-TNF were compared by qRT-PCR. The abundance of the miRNAs was normalized to abundance of U6 snRNA. **p < 0.01. Error bars represent means ± SD. Statistical analyses were performed using independent unpaired two-tailed Student’s *t*-tests.

**
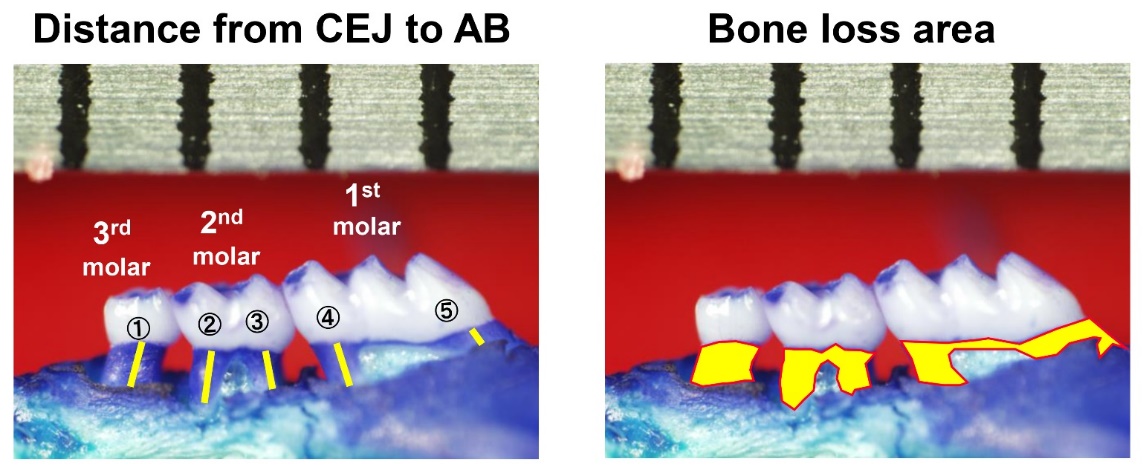
**

**Fig. S2. Schematic illustration of the measurements for periodontal bone loss.**

**
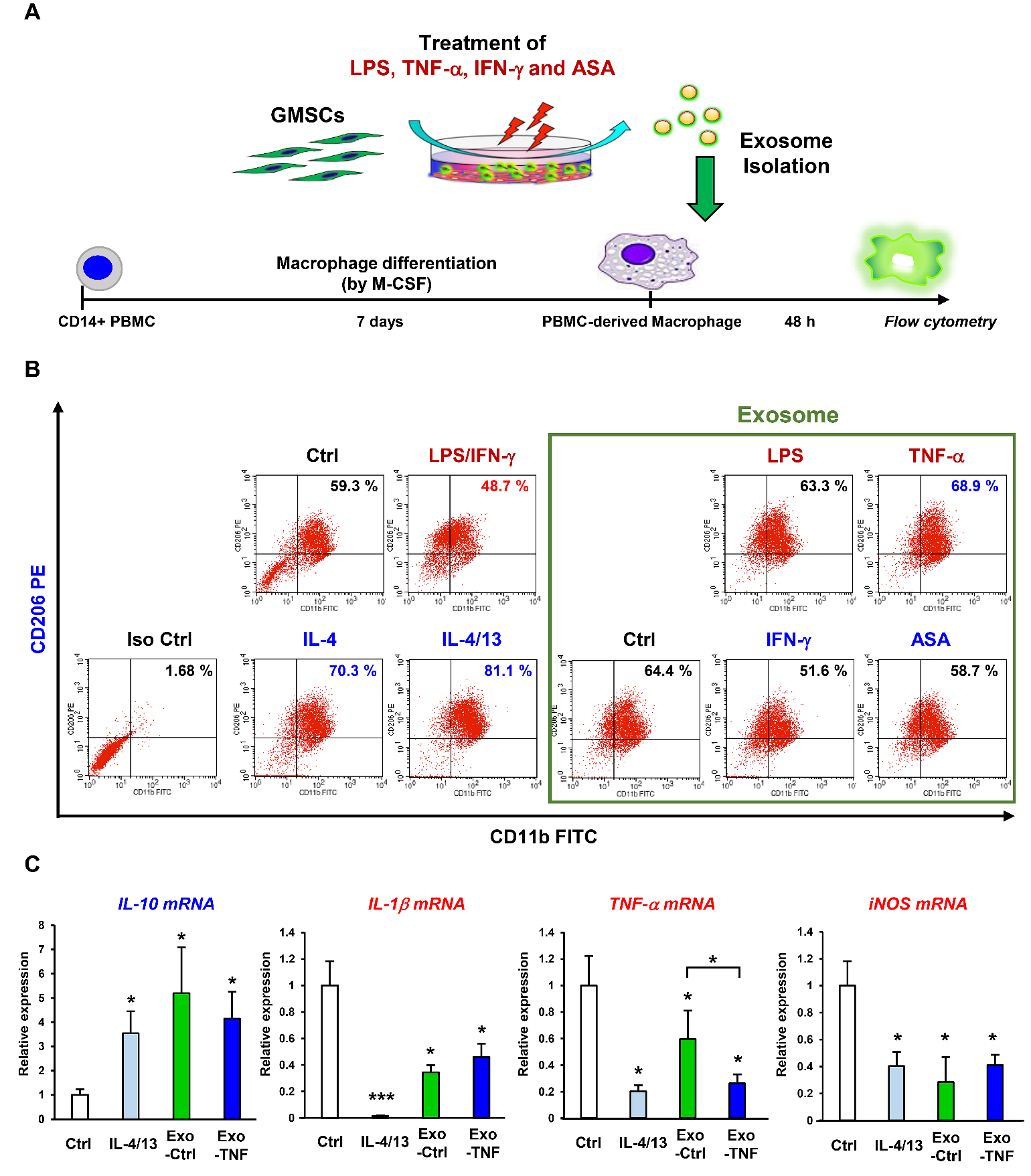
**

**Fig. S3.** (A) Experimental diagram for PBMC-differentiated macrophage stimulation using GMSC-derived exosomes. GMSCs were preconditioned with 100 ng/mL of LPS, TNF-α, IFN-γ or ASA for 48 h, and exosomes were purified from each culture medium. Macrophages were incubated with 5 μg/mL of GMSC-derived exosomes for 48 h, and the expression of M2-related CD206 marker was examined. (B) Representative FACS dot plots of CD206 expression on macrophages after stimulation to polarize toward M1/M2 phenotypes and CD14+ PBMCs were differentiated into resting macrophages by M-CSF induction (Ctrl), followed by stimulation with LPS and IFN-γ (LPS/IFN) to obtain M1 macrophages, or with IL-4 alone (IL-4), or together with IL-13 (IL-4/13) to polarize into M2 macrophages for 48 h (**left**). To validate the effects of pre-conditioned GMSC-derived exosomes on M2 macrophage polarization, differentiated resting macrophages were stimulated with exosomes for 48 h (**right**). Percentage of double positive cells (CD11b+ CD206+) was analyzed to compare M2 macrophage. (C) Comparison of cytokine mRNA expression in macrophages. Resting macrophages (Ctrl) were treated with IL4/13 and GMSC-derived exosomes with (Exo TNF) or without TNF-α (Exo Ctrl) preconditioning for 48 h. Inflammatory M1 (red) and anti-inflammatory M2 (blue) cytokine mRNA expressions were measured using qRT-PCR.**
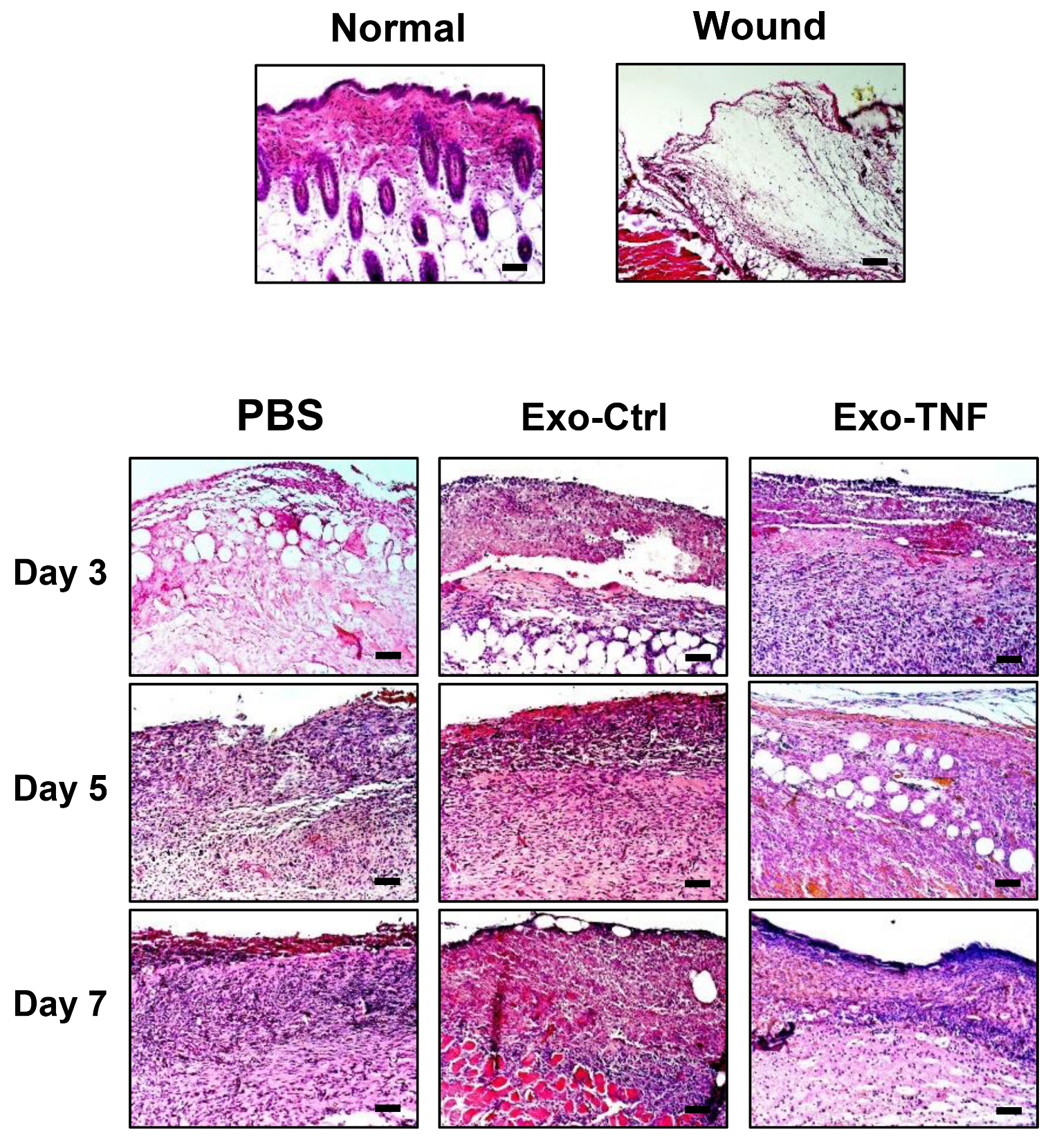
**

**Fig. S4. Therapeutic effect of GMSC-derived exosomes on skin wound healing in mice.** Representative H&E-stained paraffin-embedded sections of full-thickness incisional skin wounds in each group. Mice were sacrificed at different days post-wounding. Scale bar = 50 μm.

**
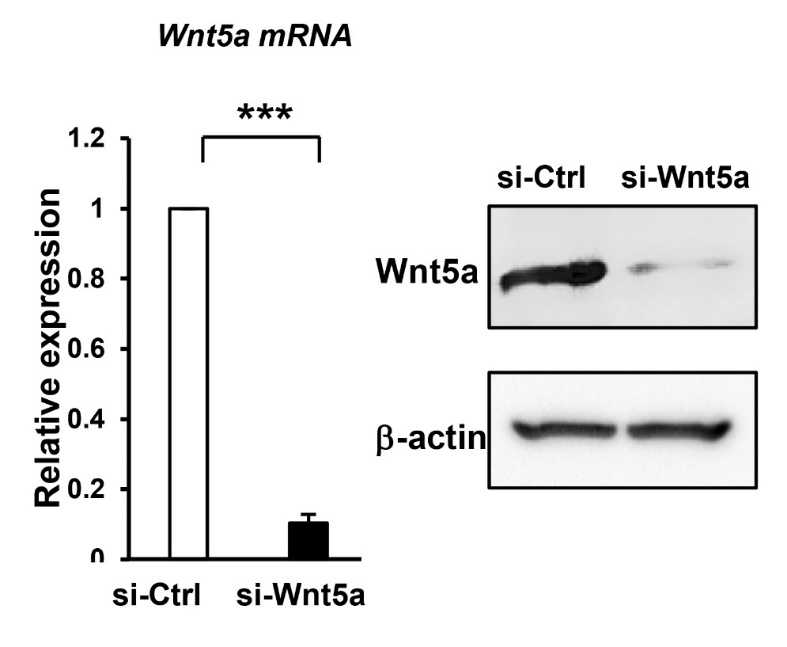
**

**Fig. S5. Knockdown of Wnt5a in PDL cells.**

PDL cells were transfected with siRNA for 24 h, and expressions of Wnt5a were validated using qRT-PCR (left) and western blotting (right). Control siRNA (si-Ctrl), Wnt5a siRNA (si-Wnt5a).

**
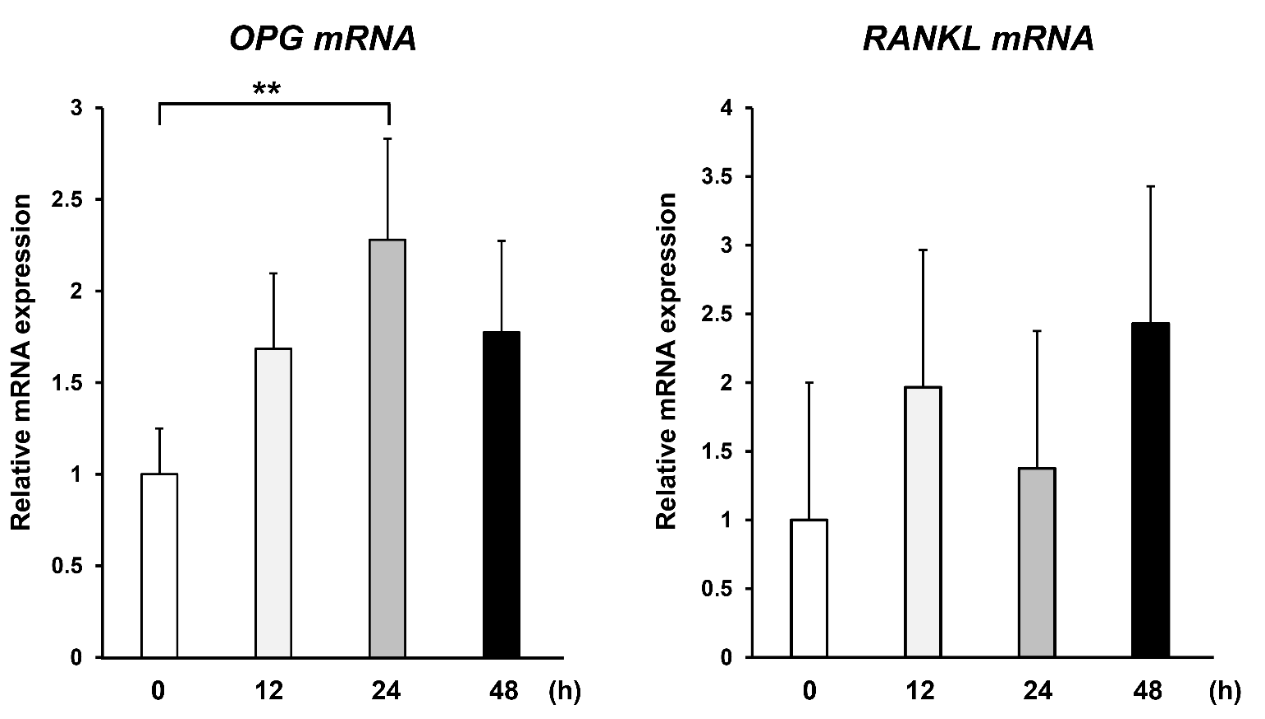
**

**Fig. S6. Effect of LPS on OPG and RANKL mRNA expression in mouse gingival fibroblast cells.** Cells were stimulated with LPS (1 μg/mL) for 12, 24, and 48 h and qRT-PCR was performed. **p < 0.01. Error bars represent means ± SD. Statistical analyses were performed using independent unpaired two-tailed Student’s *t*-tests.
